# Leveraging Machine Learning Approaches for Predicting Antidepressant Treatment Response Using Electroencephalography (EEG) and Clinical Data

**DOI:** 10.3389/fpsyt.2018.00768

**Published:** 2019-01-14

**Authors:** Natalia Jaworska, Sara de la Salle, Mohamed-Hamza Ibrahim, Pierre Blier, Verner Knott

**Affiliations:** ^1^Institute of Mental Health Research, University of Ottawa, Ottawa, ON, Canada; ^2^Cellular & Molecular Medicine, Faculty of Medicine, University of Ottawa, Ottawa, ON, Canada; ^3^Brain and Mind Research Institute, University of Ottawa, Ottawa, ON, Canada; ^4^Department of Mathematics, Faculty of Science, Zagazig University, Zagazig, Egypt

**Keywords:** major depressive disorder (MDD), antidepressants, biomarker, quantitative EEG, machine learning (ML), classification and regression trees, predictive models, personalized treatment

## Abstract

**Background:** Individuals with major depressive disorder (MDD) vary in their response to antidepressants. However, identifying objective biomarkers, prior to or early in the course of treatment that can predict antidepressant efficacy, remains a challenge.

**Methods:** Individuals with MDD participated in a 12-week antidepressant pharmacotherapy trial. Electroencephalographic (EEG) data was collected before and 1 week post-treatment initiation in 51 patients. Response status at week 12 was established with the Montgomery-Asberg Depression Scale (MADRS), with a ≥50% decrease characterizing responders (*N* = 27/24 responders/non-responders). We used a machine learning (ML)-approach for predicting response status. We focused on Random Forests, though other ML methods were compared. First, we used a tree-based estimator to select a relatively small number of significant features from: (a) demographic/clinical data (age, sex, individual item/total MADRS scores at baseline, week 1, change scores); (b) scalp-level EEG power; (c) source-localized current density (via exact low-resolution electromagnetic tomography [eLORETA] software). Second, we applied kernel principal component analysis to reduce and map important features. Third, a set of ML models were constructed to classify response outcome based on mapped features. For each dataset, predictive features were extracted, followed by a model of all predictive features, and finally by a model of the *most* predictive features.

**Results:** Fifty eLORETA features were predictive of response (across bands, both time-points); alpha_1_/theta eLORETA features showed the highest predictive value. Eighty-eight scalp EEG features were predictive of response (across bands, both time-points), with theta/alpha_2_ being most predictive. Clinical/demographic data consisted of 31 features, with the most important being week 1 “concentration difficulty” scores. When all features were included into one model, its predictive utility was high (88% accuracy). When the *most* important features were extracted in the final model, 12 predictive features emerged (78% accuracy), including baseline scalp-EEG frontopolar theta, parietal alpha_2_ and frontopolar alpha_1_.

**Conclusions:** These findings suggest that ML models of pre- and early treatment-emergent EEG profiles and clinical features can serve as tools for predicting antidepressant response. While this must be replicated using large independent samples, it lays the groundwork for research on personalized, “biomarker”-based treatment approaches.

## Introduction

Worldwide, major depressive disorder (MDD) carries a large burden of disease (1), is associated with impaired daily functioning (2), and worsening of co-morbid medical illness (3, 4). It is also linked with shorter life expectancies (5), including increased death by suicide. However, one of the largest naturalistic clinical trials assessing treatment outcomes in depressed patients found that fewer than ~50% responded (>50% symptom decreases), and only ~30% remitted (absence/near absence of symptoms), to intervention with a serotonin reuptake inhibitor (SSRI) antidepressant (6, 7); SSRIs are the most common antidepressant pharmacotherapy for treating MDD. Unfortunately, partial or inadequate response carries serious consequences, as each attempt to improve outcome either by switching or combining pharmacotherapies may require weeks to evaluate effectiveness (8, 9). This represents a substantial amount of time during which patients live with lingering, debilitating and even fatal symptoms.

Current approaches for treating MDD rely on trial-and-error sequential treatment strategies, as there is currently no established method of predicting whether a medication will lead to response. Identifying markers of response, either by *a priori* prediction or by distinguishing eventual responders from non-responders shortly after commencing treatment, would significantly increase the efficiency and efficacy of MDD interventions. Evidenced-based decision-making regarding treatment selection may be aided by biomarkers. Biomarkers are measurable and objective indicators of biological processes, or biological responses to interventions (10). To date, there have been no identified biomarkers of sufficient clinical utility to inform antidepressant treatment selection (11, 12). Nevertheless, growing evidence supporting mood disorders as brain disorders with putative structural and functional abnormalities in certain neural circuits (13) has positioned neuroimaging techniques as candidates for prognostic biomarkers in MDD (14–21), and as potential indices of treatment response prediction.

For routine clinical use, predictive biomarkers must have a high specificity/sensitivity, be reproducible, yet also be relatively inexpensive, non-invasive and accessible (22). Although not possessing the same spatial resolution as functional magnetic resonance imaging (fMRI), quantitative measures of brain electrical signals derived from scalp-recorded electroencephalograms (EEG) provide superb temporal resolution of brain activity. Further, EEG offers many of the outlined practical advantages [e.g., easy-to-administer, low-cost; (23)]. Power spectral measures of resting-state EEG activity have been found to be sensitive to both acute and chronic effects of antidepressant pharmacotherapies in MDD (24, 25). Additionally, members by our own group and others have found that when EEG profiles are assessed before or early in the course of treatment (≤1 week), they are predictive of/associated with antidepressant response (e.g., theta EEG source-localized to the anterior cingulate cortex [ACC] or alpha EEG/frontal alpha asymmetry) (26–29). Alpha power is thought to be inversely related to cortical arousal (30), therefore, excess alpha power may represent decreased cortical arousal (though alpha presence should not be thought of as reflecting an “idle”/inactive brain state). Measures of prefrontal theta cordance, which is a combination of absolute and relative EEG power, have been shown to correlate strongly with cerebral perfusion (31), and have also been associated with treatment response. In other words, theta cordance appears to be an electrocortical proxy of fronto-cortical activity as indexed by cerebral blood flow. Several groups have noted that initial (32) or rapid decreases in prefrontal theta cordance were associated with a positive response to treatment with various antidepressant pharmacotherapies (33, 34). However, these predictive EEG profiles tend to be limited to group-level comparisons, which may obscure potentially useful information at the individual-level. Importantly, individual EEG-based biomarkers would be most useful clinically.

The complexity/dimensionality of EEG data lends itself to the use of machine learning (ML) approaches which, unlike conventional analyses, are designed to deal with multivariate inputs. ML can treat EEG measures as patterns rather than considering each measure in isolation, which could potentially be a more informative analytic approach (35, 36). Further, ML approaches may be more conducive to extracting response prediction data at the individual-level (after we are sufficiently confident that we input appropriate information). While there have been several ML-based studies using EEG data to separate individuals with and without MDD (37), including work from our own group (38), there have only been a handful of studies utilizing ML-based approaches of EEG data for response prediction (see Supplementary Table 1 for a summary). However, the few that exist have yielded relatively high prediction accuracies of response to SSRI treatment based on pre-treatment EEG features (39), and appear to be more accurate than prediction models based on clinician ratings (40). A recent study of depressed individuals treated with repeated transcranial magnetic stimulation (rTMS) assessed baseline and week 1 EEG profiles, including theta and alpha power and connectivity, frontal theta cordance and alpha peak frequency (41). A ML approach was used to differentiate responders/non-responders using these measures, coupled with depression change scores. The 12 eventual rTMS responders were separated from non-responders (*N* = 30) based on elevated theta connectivity at baseline and week 1 (sensitivity: 0.84; specificity: 0.89). The same group also found that a ML model consisting of 30 features, collected during a working memory task (including baseline/week 1/changes in theta, upper alpha & gamma power, connectivity, theta-gamma coupling), could distinguish rTMS responders/non-responders [sensitivity: 0.90; specificity: 0.92; (42)]. In addition to the SSRI and rTMS findings, frontal EEG sites have been shown to be most predictive of clinical and cognitive outcome in MDD patients following transcranial direct current stimulation (tDCS) treatment using ML approaches (43).

Despite the promise shown by the application of ML for predicting antidepressant treatment response, logistical obstacles exist (e.g., ethics/privacy concerns, technical expertise). Other challenges which impede ML from being used in predictive psychiatry include the relatively small sizes (though that is not an inherent limitation *per se*) of many clinical/biological datasets. A further challenge is that that data may be lacking/are incomplete, or datasets require considerable processing prior to analyses (therefore, the preparation of the data for analysis can be onerous). Further, ML sometimes focuses on the most efficient use of data rather than the most valuable, which leads to variability in ML approaches (including biases), and the tendency to overfit data (44). Additionally, many studies using ML on EEG data in antidepressant response prediction tend to be based on unequal responder/non-responder samples, which requires over/under sampling techniques (e.g., Synthetic Minority Oversampling Technique [SMOTE] or weighting subjects by their inverse proportion of being responders or non-responders); whether this is applied is generally not stated in the methodology. Under-sampling may lead to discarding potentially useful data while over-sampling duplicates samples, which could greatly increase the possibility of overfitting. As a result, ML-derived results can sometimes be difficult to replicate, and comparisons between various ML approaches in one study are rare. We are also not aware of any studies that have assessed whether source-localized EEG activity using approaches such as low-resolution brain electromagnetic tomography [LORETA; (45)] contribute to antidepressant response prediction with ML, despite the fact that this has shown predictive promise using non-ML analyses. Finally, the contribution of specific depression symptom scores [e.g., items of the Montgomery-Asberg Depression Rating Scale [MADRS]; (46)] and demographic features (e.g., age, sex), are generally not included in ML approaches utilizing EEG data for predicting response. This is despite the fact that specific symptom profiles and demographic variables have been shown to be predictive of response (47).

As such, in the present study we carried out several experiments addressing the outlined gaps. First, we explored the utility of exact low-resolution electromagnetic tomography software (eLORETA)-localized EEG data at baseline and at week 1 of antidepressant pharmacotherapy in predicting responder (*N* = 27)/non-responder (*N* = 24) status (i.e., balanced sample) in depressed adults at week 12 of treatment using several ML approaches, with a focus on Random Forest. In other words, we extracted predictive features of response from eLORETA data (**Experiment A**). Second, the same ML approaches were applied to scalp-level EEG power in order to extract pertinent predictive features from this dataset (**Experiment B**). Third, ML was applied to clinical and demographic data to extract predictive features of response (i.e., sex, age, individual/total MADRS score items at baseline/week 1, score changes; **Experiment C**). Subsequently, all of the relevant predictive features were put into a combined ML model, and prefrontal theta cordance data was included (**Experiment D**), this was followed by a final analysis that identified the *most* relevant predictive features of antidepressant response (**Experiment E**). We expected that ML approaches would be useful for predicating antidepressant response, and that the combined model would yield superior prediction values as compared to each individual model.

## Methods

### Participants

In total, 51 adults (18–60 years) with a primary diagnosis of MDD, and enrolled in a clinical trial assessing antidepressant pharmacotherapies [details below; (48)], participated in this EEG study. As previously outlined (49), psychiatrists ascertained the diagnosis with the Structured Clinical Interview for DSM (Diagnostic & Statistical Manual of Mental Disorders) IV-TR Diagnoses, Axis I, Patient Version [SCID-I/P; (50)]. Symptom severity was evaluated using the MADRS (46), with scores ≥22 at enrollment. A secondary diagnosis of an anxiety disorder was permitted. Patients with Bipolar Disorder (BP I/II or NOS), psychosis history, current (<6 months) drug/alcohol abuse or dependence, history of seizures, unstable (≥3 months) medical condition(s) and history of anorexia/bulimia were excluded. Patients were not taking psychoactive drugs at the time of randomization, and appropriate drug washout periods were applied prior to enrollment. EEG testing occurred pre- and 1-week post-treatment. Participants provided written informed consent, and the study was approved by the Royal Ottawa Health Care Group Research Ethics Board.

### Clinical Trial Design

As part of a larger clinical trial (48), patients were randomized (double-blind) to one of three antidepressant regimens: escitalopram + bupropion (ESC+BUP), escitalopram (ESC) + placebo or bupropion (BUP) + placebo. Treatments were initiated at recommended starting doses, and raised only if tolerated. MADRS assessments were conducted prior to treatment, weekly for the first 4 weeks, and then bi-weekly until week 12. Change in MADRS scores from baseline to week 12 were used to categorize patients as responders (*N* = 27; ≥50% MADRS score reduction) or non-responders (*N* = 24; < 49% MADRS score reduction). Responder groups were similar on demographic and clinical parameters at baseline (Table 1).

**Table 1:** Clinical characteristics and demographics of participants (Means ± S.D.).

	**All**	**Treatment**	**Treatment**
	**participants**	**responders**	**non-responders**
	**(*N* = 51)**	**(*N* = 27)**	**(*N* = 24)**
Sex (M/F)	24/27	12/15	11/13
Age	40.2(±11.8)	35.9(±11.3)	45(±10.6)
Total baseline MADRS scores	30.6(±5.2)	29.6(±4.5)	31.6(±5.8)
Total week 1 MADRS scores	26.2(8.7)	22.7(7.9)	30.2(7.9)
Total week 12 MADRS scores	15.9(12.5)	6.2(5.2)	26.8(8.6)
% Change (week 1-baseline)	−13.7(27.5)	±23.2(24.6)	−3.1(27.1)
% Change (week 12-baseline)	−48.8(37.9)	−79.0(17.0)	−14.9(22.9)

*MADRS, Montgomery-Asberg Depression Rating Scale*.

### EEG Recordings and EEG Data Processing

As described elsewhere (49), prior to each EEG session, participants abstained for >3 h from caffeine and/or nicotine, as well as from alcohol/drugs (excluding prescribed drugs) as of midnight. Using an average scalp reference, AF_z_ ground, and a sampling rate of 500 Hz, EEG recordings were obtained from 32 sites using the 10–20 system (see Supplementary Figure 1) by way of Ag/AgCl electrodes embedded in a cap (EasyCap, Inning A. Ammersee, Germany). Additional electrodes were used to monitor vertical and horizontal electrooculographic (EOG) activity. Amplifier filters were 0.1–80 Hz, and electrode impedance was ≤5 kΩ during recordings (Brain Vision Quickamp®; Brain Products, Gilching, Germany). Vigilance-controlled resting-state EEG activity was recorded for 3 min during the eyes-closed (EC) condition (BrainVision Recorder®, Brain Products, Gilching, Germany).

EEG processing included re-referencing with averaged mastoid electrodes (TP_9/10_), filtering (0.1–30 Hz) and segmentation (2 s; Brain Vision Analyzer® Software, Brain Products, Gilching, Germany). Ocular-corrected [Gratton & Coles method; (51)] epochs were excluded if voltages exceeded ±75 μV. A minimum of 100 s of artifact-free data were subjected to a Fast Fourier Transform algorithm (Hanning Window; 5% cosine taper) for computation of absolute (μV^2^) power in frequency bands of interest (delta: 1–4 Hz, theta: 4–8 Hz, alpha_1_: 8–10.5 Hz, alpha_2_: 10.5–13 Hz, beta: 13–30 Hz) at 28 sites (mastoids, ground, reference electrode sites excluded). EEG data was ln-transformed prior to analyses to ensure normality. The ln-transformation minimizes the influence of extreme values (i.e., skewness) within the dataset.

### eLORETA Analyses

EC ln-normalized EEG data in each frequency band (mastoid-referenced) was subjected to analysis with eLORETA (45) software (v. 2081104). eLORETA analysis estimates neural activity as current density based on the Montreal Neurological Institute-152 template creating a low-resolution activation image. The solution space consists of 6,239 voxels (5 mm^3^ voxel) restricted to gray matter. Current source density is calculated from a linear, weighted sum of scalp potentials. This value is then squared per voxel, yielding current density power measures (A/m^2^). Its validation has been independently replicated (52), and cross-validated (53, 54). Current source density measures from 84 Brodmann areas (BA; 42/hemisphere), available through the eLORETA software, were extracted (single voxel at the centroid of each BA).

### Theta Cordance Analyses

Theta EEG cordance values were calculated using an algorithm provided by the UCLA Laboratory of Brain, Behavior, and Pharmacology (55). Briefly, values were computed by normalizing theta power across electrode sites (calculated using 19 electrodes, 30 bipolar pairings) and then combining absolute and relative theta power values. Average cordance values from prefrontal electrodes (Fp_1_, Fp_2_) at baseline and week 1 were extracted, as these two sites have been shown to be most predictive of response outcome in the past (33, 34).

### Machine Learning (ML) Methodology

As outlined, patients were classified into responders (*N* = 27) and non-responders (*N* = 24, i.e., this was the dependent/outcome feature) based on their clinical outcome by week 12 (Table 1), thus, this ML problem was a binary classification problem. To achieve our objective, which was to assess the utility of the datasets for predicting week 12 response, we started by pre-processing the data.

#### Data Preprocessing

Initially, we prepared and structured the raw data in order to obtain the final datasets that could be used to build predictive ML models. The preprocessing of data consisted of the following steps:

- Construction of Analytical Base Tables
  We constructed the following analytical base tables (ABT): (1). ln-normalized absolute EEG power from 28 electrodes (mastoid-referenced/EC data) at baseline and after week 1 of treatment for each of delta, theta, alpha_1_, alpha_2_, and beta bands. (2). eLORETA-localized power values (ln-normalized mastoid-referenced/EC data) at 84 BAs at baseline and week 1 for each band. (3). theta cordance data (EC data from left and right prefrontal sites) at baseline and week 1. (4). clinical/demographic data consisting of age, sex, each item of the MADRS (10 items) as well as total MADRS scores at baseline and week 1 as well as change scores for each MADRS item (i.e., difference from baseline to week 1).
- Data Clean Up
  Individuals with missing data (i.e. those without week 1 data) were removed from the final ATB tables (*N* = 2). As such, the final sample per ATB table included *N* = 27 responders and *N* = 24 non-responders (*N* = 51 total).
- Data/Feature Scaling & Normalization
  Subsequently a scaling technique, called Min-Max scaling (56), was applied to normalize the data (also referred to as data features or attributes) to a fixed range between minimum and maximum values. Given a feature/attribute “A,” the Min-Max scaling value *x*_*norm*_ of a value *x* in “A” is done via the following equation:

$$\begin{array}{l} x_{norm}=\left(\frac{x-x_{min}}{x_{max}-x_{min}}\right)\times \left({\overset{´}{x}}_{max}-{\overset{´}{x}}_{min}\right)+\;{\overset{´}{x}}_{min} \end{array}$$

Where *x*_*min*_ and *x*_*max*_ are the minimum and maximum values in features “A” respectively, and ${\overset{´}{x}}_{min}$ and ${\overset{´}{x}}_{max}$ are the new minimum and maximum values of “A” after scaling. As such, if ${\overset{´}{x}}_{min}=0$ and ${\overset{´}{x}}_{max}=1$, then the maximum absolute value of “A” is scaled to unit size. In practice, scaling plays an important role for improving predictive models' performance (57, 58). The motivation to use this form of data standardization is due to its robustness to small standard deviations of features, and preserving zero (or near zero) entries in relatively sparse datasets. Further, standardization brings all features into the same range, allowing for scale-invariant features. Generally, ML algorithms benefit from data standardization to efficiently reduce data dimensionality, which aids with learning algorithms and prediction.

#### Machine Learning Strategy: Three Stages

Although there are numerous ML approaches we could have adopted, we focused on results obtained using Random Forests (RF). As such, all of the steps are described in relation to RF.

- Stage 1: Tree-Based Feature Selection
  As the large number of features involved in the structured ABTs (i.e., datasets) can represent a bottleneck for building efficient predictive models, we applied the extremely randomized trees (ERT) algorithm (59) to simplify the ABTs by discarding irrelevant features. Irrelevant features frequently capture unnecessary/redundant and noisy data. ERT is a tree-based feature selection algorithm that can be used to rank features using an importance measure (e.g., average Gini impurity reduction score). Relevant features are obtained by discarding irrelevant features that have an importance score less than a certain threshold (e.g., average impurity reduction ≥0.01). Strictly speaking, ERT builds an ensemble of unpruned decision trees and aggregates their outputs for prediction. When building each decision tree in ERT, every node uses Gini impurity measures (60) as a locally optimal condition on a single feature to split the ABT into two subsets such that the samples with identical classes (i.e., target value – in this case, responders and non-responders) end up being in the same subset. Gini impurity measure can be computed as follows:
  $$\begin{array}{l} Gini\text{\_}impurity\left(ABT\right)=\overset{K}{\underset{i=1}{\sum }}p_{i}\;\times \;\left(1-p_{i}\right) \end{array}$$
  Where *K* is the number of class labels (target values: responders/non-responders), and *p*_*i*_ is the probability of a certain classification *i* in *K*. Thus, *G*(*ABT*) measures the likelihood of an incorrect classification of a new sample of a random feature, if that new instance were randomly classified according to the distribution of class labels from the dataset. Thus, during the training of each tree, we can quantify how each feature decreases the weighted impurity in this tree (i.e., with every split made of a node on a feature, the Gini impurity measures of the two descendent nodes should be less than the parent node). Thus, averaging the Gini impurity reduction for each single feature over all trees in the forest provides its importance, which allows us to rank the features based on their relevance. It is worth noting that using only a relatively small number of important features can dramatically enhance the generalization of the constructed predictive models (i.e., classifiers) by reducing overfitting. This is why we ran feature selection per dataset (vs. on all of the data).
- Stage 2: Feature Mapping
  We then applied kernel principal component analysis (KPCA) on the purified ABTs after removing irrelevant features to map relevant features and reduce dimensionality (61, 62). That is, KPCA is a method that uses a kernel function κ to project the important features data onto a new space. This space often contains a small number of features (compared to the original datasets) and where the samples in the purified ABTs become linearly separable and can be discriminated by finding a decision between the given classes (i.e., responders/non-responders) in the newly mapped space that best maximizes class separation.
  In KPCA, the kernel is a nonlinear function κ such that for all samples *x*_*i*_, *x*_*j*_∈ purified ABT, we have that κ〈φ(*x*_*i*_), φ(*x*_*j*_)〉, where φ is a mapping from purified ABTs to an inner product feature space (e.g., dot product space). While several kernel functions can be used, such as polynomial and sigmoid, we frequently obtained our best results when applying the radial basis function (RBF) Gaussian kernel, which can be calculated on two samples as follows: $\langle x_{i}{,x}_{j}\rangle =\;exp\left\{-\;\frac{||x_{i}-x_{j}|{|}^{2}}{2\;\sigma^{2}}\right\}\;$, where σ is a free parameter. Thus, in the current work, we used the RBF.
  Other commonly used feature mapping methods, such as Linear Discriminant Analysis (LDA) and standard PCA, often only allows linear dimensionality reduction. Thus, if the data has more complicated structures, which normally cannot be represented in a linear subspace, such methods will produce poor mapping. Thus, one key advantage of using KPCA in the current work is that it allows us to generalize standard PCA to nonlinear dimensionality reduction (63) and could therefore provide efficient mapping of complicated data.
- Stage 3: Predictive Data Modeling
  At this point, the purified mapped ABT datasets could be classified by building classification models, such as Random Forest [RF; (64, 65)]. RF is a tree-based ensemble learning method that operates by constructing a forest of decision trees at the training phase. That is, we used the mapped purified ABTs to create a number of decision trees. For each sample, RF aggregates the predicted class labels (responder or non-responder) of the individual trees. It then performs a mode vote among all trees to produce the final class prediction. In RF, we created a number of decision trees (i.e., estimators) in the forest in the domain of {10, 50, 100, 500}. Since we obtained slightly better classification error rates when using approximately 100 estimators this was what was used in the current study [i.e., where the asymptote in the error rate reduction occurred; (66)]. In other words, the classification error rates stabilized with ~100 decision trees (no notable improvement was noted with 500 decision trees), which is consistent with what others have suggested for RF (67). Since we have a forest of decision trees to be trained, we considered the best random split using the Gini measurement. We chose a minimum impurity split of zero for early stopping of the tree growth (64). We conducted a 10-fold cross validation to increase the accuracy of the classification process and applied regularization methods within our classification models. Summary of the best parameter values that were obtained for each ML method and dimensionality of data matrices can be found in Supplementary Tables 5 and 6, respectively.

#### Experimental Evaluation

We ran three experiments for RF classifier on each of the following purified mapped ABTs: (a) eLORETA dataset (**Experiment A**); (b) EEG dataset (**Experiment B**); (c) clinical/demographic dataset (**Experiment C**). Subsequently, all relevant features were combined into one predictive ML model that also included the cordance dataset, which, given the low-dimensionality of the data, did not undergo feature selection (**Experiment D**). This was followed by a model extracting the *most* predictive features of response/non-response (**Experiment E**).

In order to guarantee a robust study/compare other ML approaches, we additionally explored the following prominent ML predictive models: (1). Classification and Regression Tree [CART; (40, 68, 69)]; (2). Support Vector Machine [SVM; (70)]; (3). Adaboost (71); (4). Multilayer Perceptron (MLP) (72); and (5). Gaussian Naïve Bayes (73). Please see Supplementary Information for further details on these methodologies.

All ML predictive models were implemented, learned and tested using Python programming language and Scikit-learn toolkit package on an Intel(R) Core(TM) i7-2600 CPU @ 3.20 GHz computer with 16 GB of memory running on Windows 10.

#### Experimental Setting

We trained RF models using a 10-folds cross-validation for predicting response (responder/non-responder) on the underlying sub-datasets. That is, in the training phase, we iteratively learned the parameters of models using nine out of the 10-folds in the sub-dataset. Additionally, and to avoid overfitting, we applied regularization methods within our classification models by adding penalty terms for extreme parameters in their objective functions. Specifically, we pruned the tree in RF (and also CART classifier) by penalizing the selection of features and limiting the maximum allowable tree depth (we used L2-norm regularization for SVM).

#### Evaluation Metrics

In order to judge the performance of the ML classification algorithms, during the testing phase, and using the learned models, we carried out response prediction on all patients by conducting the following: We ran all algorithms until convergence, and then recorded their confusion matrices on the leave-out fold by calculating: (1). The proportion of responders that were correctly classified (i.e., true positives [TP]); (2). The proportion of non-responders that were correctly classified (i.e., true negatives [TN]); (3). The proportion of responders that were misclassified as non-responders (i.e., false positive [FP]); and (4). The portion of non-responders that were misclassified as responders (i.e., false negative [FN]). Based on such confusion matrices, we compared the accuracy of all tested ML predictive models by computing the following evaluation metrics: (a) *Receiver Operating Characteristic (ROC) Curves* (74)—These plot the true positive rate (TPR or sensitivity/recall = $\frac{TP}{TP+FN}$: the probability of the correct identification of the presence of a disorder) against the true negative rate (TNR or specificity = $\frac{TN}{TN+FP}$: the probability of the correct identification of the absence of a disorder) at various thresholds. The closer the ROC curve is to the diagonal, the less accurate the prediction. Thus, a ROC is commonly used as a robust metric to compare diagnostic accuracy of classification methods. (b) *Average F1-Score* (75)—This score measures the harmonic mean of recall and positive predictive value (PPV or precision = $\frac{TP}{TP+FP}$: the probability that the presence of a disorder in a given patient is correctly identified). In this case, if the prediction probability is >0.5, then the person is predicted to be a responder, otherwise, the person is predicted to be a non-responder. F1-scores are insensitive to FN, and therefore, it quantifies the quality of an algorithm for predicting the true positives. (c) *Area Under the Curve (AUC)*-In the context of the current study, AUC quantifies the overall ability of the classification model to discriminate responders/non-responders (76). The greater the area under the curve (i.e., closer it is to 1), the more accurate the prediction (chance is 0.5). Additional evaluation metrics which were computed were the negative predictive value (NPV) = $\frac{TN}{FN+TN}$: the probability that the absence of a disorder in a given patient is correctly identified, as well as overall accuracy $=\frac{TP+TN}{TP+TN+FP+FN}$.

## Results

Characteristics of the entire patient sample as well as responders/non-responders are summarized in Table 1.

### Experiment A: eLORETA ML Predictive Results

Feature selection indicated that the most predictive features of week 12 response/non-response using eLORETA data were: 6 features wherein source-localization was specific to delta, 10 to theta, 13 to alpha_1_, 9 to alpha_2_, and 12 beta. The average impurity reduction score of ≥0.01 was used to determine the importance of a feature (for all bands) in the eLORETA dataset. *Delta*: With respect to predictive delta eLORETA features, they were largely at baseline, right-localized and diffuse (though not prefrontal). Baseline delta localized to the right lingual gyrus was the most predictive delta feature. *Theta:* Predictive theta features were from baseline and localized to the occipital cortex (lingual gyri), and week 1 theta localized largely to left-lateralized temporo-parietal regions. The most predictive feature was week 1 theta localized to the left transverse temporal gyrus. *Alpha*_1_: With respect to alpha_1_ (alpha_1/2_ was split based on previous research that each band could be associated with response (49, 77), predictive alpha_1_ features were largely week 1, left-lateralized and relatively diffuse (though largely temporal). Although, baseline prefrontal alpha_1_ was also found to be a predictive feature. The most predictive feature was week 1 alpha_1_ localized to the left transverse temporal gyrus. *Alpha*_2_: Alpha_2_ predictive features were largely baseline, and localized to the left parahippocampal gyrus, right pre/frontal regions (as well as right subcallosal gyrus and ACC). The most predictive feature was baseline alpha_2_ localized to the right subcallosal gyrus. *Beta*: Predictive beta features were largely week 1 and localized to the left precuneus and precentral gyrus as well as bilateral posterior cingulate cortex, though baseline left-frontal beta was also a predictive feature. The most predictive feature was week 1 beta localized to the left precuneus (Supplementary Table 2 and Supplementary Figure 2). As evidenced by F1 scores (focus on RF), collectively, eLORETA features in the alpha_1/2_ bands were most predictive (across ML approaches), followed by theta; eLORETA-localized activity in beta/delta were less predictive of week 12 response status (Table 2).

**Table 2:** F1 scores of classifiers of source-localized (eLORETA) electroencephalographic (EEG) band power and associated area under the curve values (M ± S.D.) for random forest.

	**Alpha_**1**_**	**Alpha_**2**_**	**Beta**	**Delta**	**Theta**
Random forest	0.752	0.803	0.674	0.682	0.692
(AUC values)	(0.75 ± 0.22)	(0.74 ± 0.20)	(0.62 ± 0.19)	(0.69 ± 0.25)	(0.77 ± 0.21)
Adaboost	0.694	0.748	0.648	0.661	0.725
SVM	0.757	0.695	0.507	0.659	0.690
CART	0.635	0.638	0.591	0.569	0.659
MLP	0.749	0.585	0.536	0.672	0.771
Gaussian naive bayes	0.756	0.619	0.497	0.718	0.629

*AUC, area under the curve; CART, classification and regression trees; MLP, multilayer perceptron; SVM, support vector machine*.

### Experiment B: EEG ML Predictive Results

Feature selection indicated that the most predictive features of week 12 response/non-response using surface-level EEG power were: 17 delta EEG features, 20 theta EEG features, 14 alpha_1_ EEG features, 20 alpha_2_ EEG features, and 17 beta EEG features. The average impurity reduction score of ≥0.02 was used to determine the importance of a feature (for all bands) in the EEG dataset. *Delta*: Regarding EEG delta features, those associated with response prediction were largely at week 1, right-localized and diffuse, with a handful of predictive features at baseline (which were also predictive at week 1). The most predictive features were EEG delta power at week 1 at T_8_ followed by power at CP_6_. *Theta*: Predictive baseline EEG theta features were generally frontal and occipital; week 1 predictive EEG theta features were diffuse, though not occipital. The most predictive features were baseline EEG theta power at Fp_2_ and week 1 EEG theta power at FC_2_. *Alpha*_1_: With respect to EEG alpha_1_, predictive features were predominantly baseline and frontal. The most predictive EEG alpha_1_ feature was baseline power at F_7/8_. *Alpha*_2_: Baseline EEG alpha_2_ predictive features were diffuse, while week 1 alpha_2_ predictive features were parietal and occipital. The most predictive EEG alpha_2_ features were baseline power at P_8_ and week 1 power at O_1_. *Beta*: Finally, predictive EEG beta features existed at both baseline and week 1, and were diffuse. The most predictive features were baseline EEG beta power at T_7_ and week 1 power at Fz (Supplementary Table 3, Supplementary Figure 3, and Table 3). As evidenced by F1 scores (focus on RF), overall, features in EEG alpha_2_ (followed by theta) were most predictive of response (across ML approaches) while beta/delta were least predictive (Table 3).

**Table 3:** F1-scores of classifiers of electroencephalographic (EEG) band power and associated area under the curve values for random forest (M ± S.D.).

	**Alpha_**1**_**	**Alpha_**2**_**	**Beta**	**Delta**	**Theta**
Random forest	0.721	0.783	0.701	0.676	0.727
(AUC values)	(0.70 ± 0.2)	(0.80 ± 0.23)	(0.67 ± 0.29)	(0.72 ± 0.22)	(0.71 ± 0.34)
Adaboost	0.674	0.775	0.643	0.576	0.752
SVM	0.612	0.768	0.521	0.657	0.691
CART	0.624	0.757	0.595	0.560	0.680
MLP	0.653	0.689	0.533	0.589	0.664
Gaussian naive bayes	0.719	0.697	0.599	0.646	0.718

*AUC, area under the curve; CART, classification and regression trees; MLP, multilayer perceptron; SVM, support vector machine*.

### Experiment C: Clinical/Demographic ML Predictive Results

Feature selection indicated that there were 31 predictive features of response/non-response using demographic and clinical data. The average impurity reduction score of ≥0.02 was used to determine the importance of a feature. Age and sex were found to be predictive features (with comparable predictive value). Baseline, week 1 and score changes (week 1-baseline) were all predictive features for MADRS items #2 (sadness), #5 (reduced appetite), #6 (concentration difficulty), #8 (inability to feel), #9 (pessimistic thoughts), #10 (suicidal thoughts) and total MADRS score. Change scores were also predictive for items #1 (apparent sadness), #3 (inner tension), #4 (reduced sleep) and #7 (lassitude), as were week 1 scores for #1 and #7, as well as baseline scores for #3 and #4. Interestingly, the strongest feature predictive of response, by far, was the “concentration difficulty” score (MADRS #6) at week 1, followed by “sadness” score (#2) changes, and total MADRS score (Supplementary Table 4, Supplementary Figure 4 and Table 4).

**Table 4:** F1-scores of classifiers of clinical and demographic data as well as associated area under the curve values for random forest (M ± S.D.).

Random forest	0.737
(AUC values)	(0.74 ± 0.23)
Adaboost	0.715
SVM	0.620
CART	0.652
MLP	0.544
Gaussian naive bayes	0.534

*AUC, area under the curve; CART, classification and regression trees; MLP, multilayer perceptron; SVM, support vector machine*.

### Experiment D: Combined ML Models

**Figure 1:**
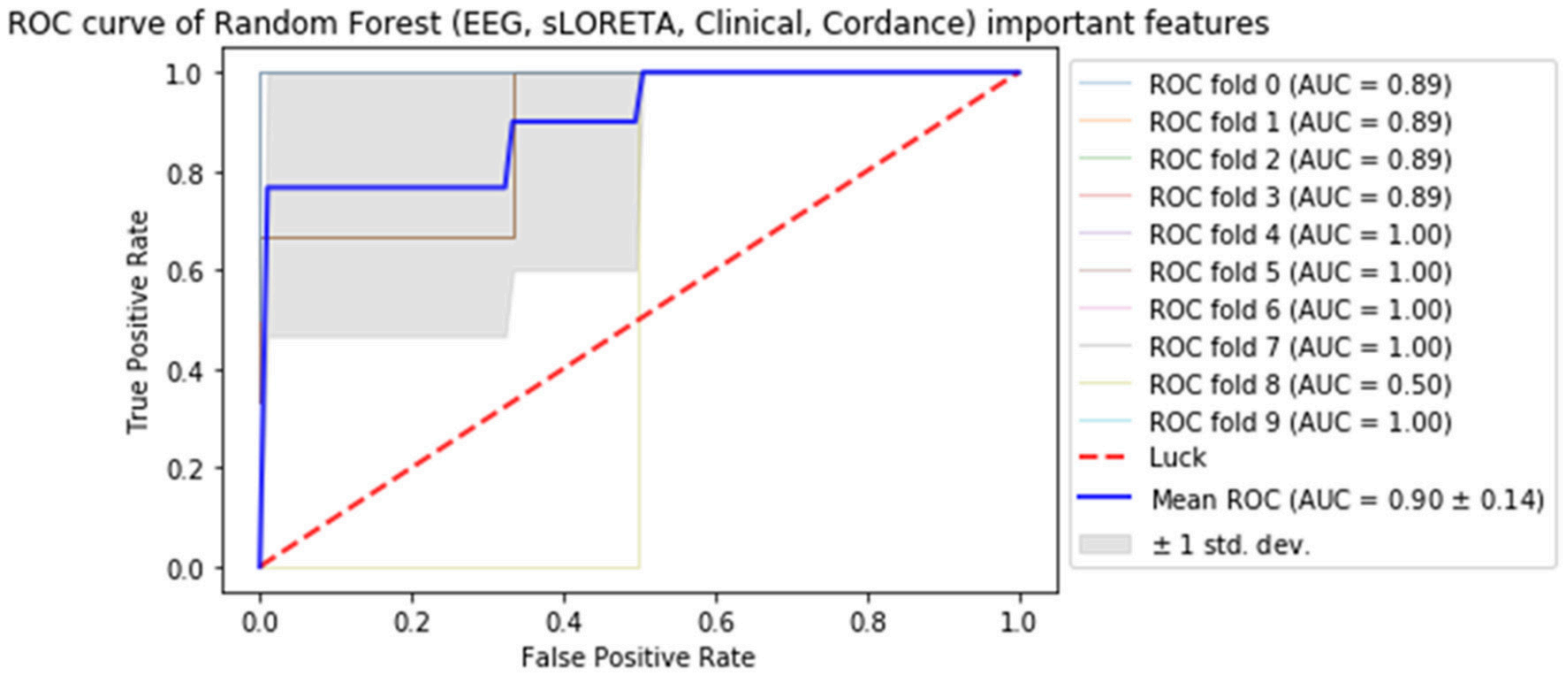
Receiver operator curve (ROC) & area under the curve (AUC) scores for all important features extracted from all datasets (source-localized EEG current density, scalp-level EEG power, clinical/demographic data & theta cordance) random forest.

Subsequently, all of the most predictive features from the above sections (**Experiments A–C**) were included in another ML experiment, to which cordance data (theta EC from baseline and week 1) was included, and the F1 values are presented in Table 5 (Figure 1). At a sensitivity of 0.77 and specificity of 0.99, the model has a PPV of 0.99, NPV of 0.81, and overall classification accuracy of 0.88.

**Table 5:** F1-scores of classifiers of important features extracted from source-localization (eLORETA) and surface-level EEG power in various bands, demographic/clinical as well as cordance data.

Random forest	0.901
(AUC values)	(0.90 ± 0.14)
Adaboost	0.838
SVM	0.716
CART	0.791
MLP	0.687
Gaussian naive bayes	0.775

*Associated area under the curve values for random forest (M ± S.D.) are presented*.

*AUC, area under the curve; CART, classification and regression trees; MLP, multilayer perceptron; SVM, support vector machine*.

### Experiment E: ML Model of Most Important Features

**Figure 2:**
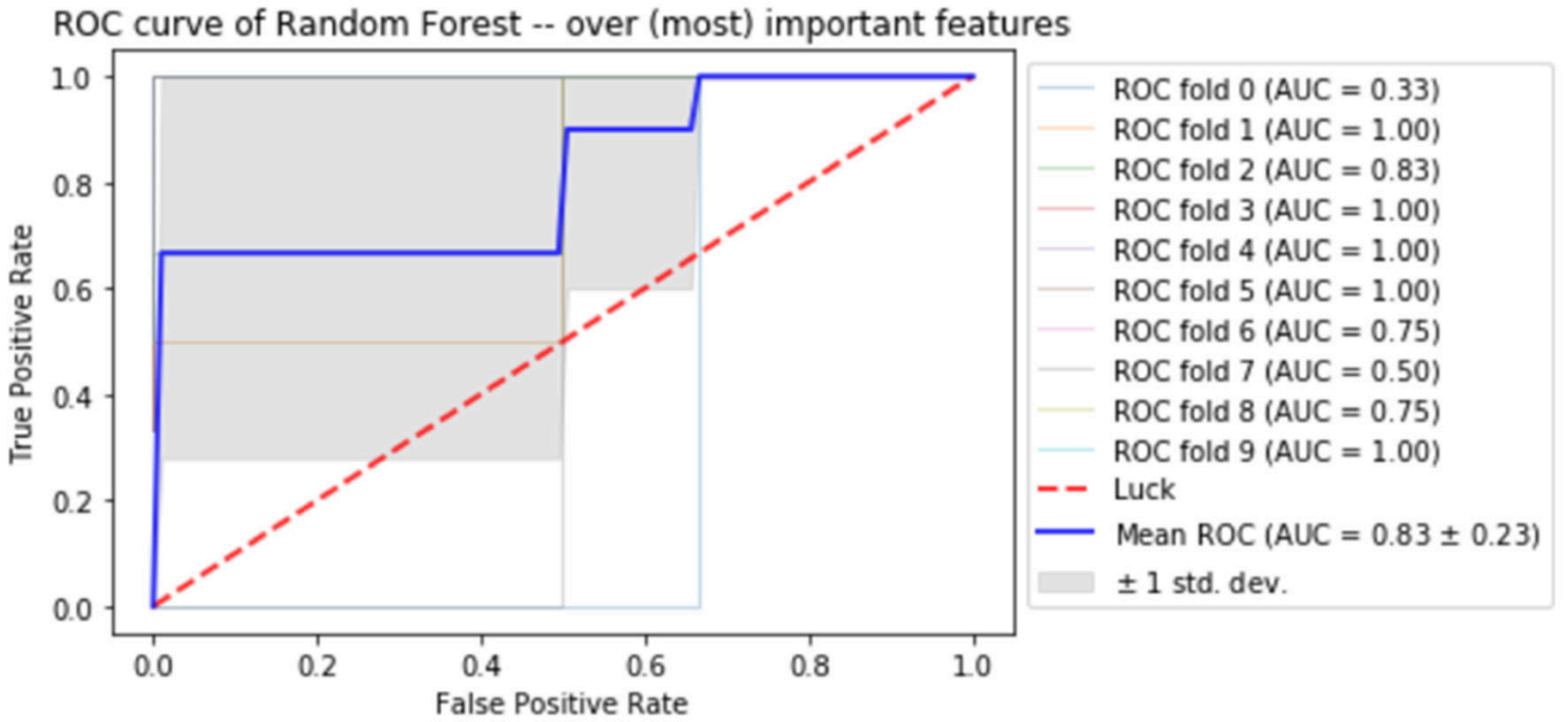
Receiver operator curve (ROC) using random forest when only the most important features are imputed into the model.

Finally, the last step was to combine all of the predictive features from above, and extract the most predictive features of response. The average impurity reduction score of ≥0.01 was used to determine the importance of a feature. As is evident from Table 6, baseline alpha_1_ power in frontopolar electrodes, baseline alpha_2_ in the right parietal electrode as well as lower frequency (delta/theta) power at the right parietal electrode at week 1 were significant features associated with response. With respect to the eLORETA data, baseline alpha_2_ localized to the ACC as well as week 1 alpha_1_/theta data localized to left temporal/auditory region were the features which most strongly contributed to response. Finally, concentration difficulties at week 1 and change in reported sadness from baseline to week 1 were the clinical features associated with response. The most predictive feature within this model was baseline theta EEG power at Fp_2_, followed closely by baseline EEG alpha alpha_2_ at P_8_, and by baseline EEG alpha_1_ power at Fp_2_. Together, these 12 features strongly predicted response status as exemplified by the F scores (Tables 6, 7 and Figure 2). At a sensitivity of 0.65 and specificity of 0.99, the model has a PPV of 0.98, NPV of 0.74, and an overall classification accuracy of 0.78.

**Table 6:** Features most predictive of antidepressant response laid out in order of importance (as indexed by average impurity reduction scores; with the top-most features having the most impact on the predictive values).

**Dataset**	**Features (in order of importance)**
EEG	Baseline Fp_2_ theta
	Baseline P_8_ alpha_2_
	Baseline Fp_2_ alpha_1_
eLORETA	Baseline alpha_1_ localized to the right subcallosal gyrus (BA25)
Clinical	Concentration difficulties at week 1
EEG	Week 1 P_8_ theta
eLORETA	Week 1 alpha_1_ localized to the left middle temporal gyrus (BA21)
eLORETA	Week 1 alpha_1_ localized to the left transverse temporal gyrus (BA41)
EEG	Week 1 P_8_ delta
Clinical	Reported sadness change score (baseline to week 1)
EEG	Baseline Fp_1_ alpha_1_
eLORETA	Week 1 theta localized to the left transverse temporal gyrus (BA41)

**Table 7:** F1-scores of classifiers of the most important features across all of the datasets and associated area under the curve values for random forest (M ± S.D.).

Random forest	0.827
(AUC values)	(0.83 ± 0.23)
Adaboost	0.815
SVM	0.730
CART	0.762
MLP	0.625
Gaussian naive bayes	0.731

*AUC, area under the curve; CART, classification and regression trees; MLP, multilayer perceptron; SVM, support vector machine*.

## Discussion

This study aimed to assess the utility of pre-treatment and week 1 clinical information as well as various types of EEG data (source-localized current density, scalp-level power, prefrontal theta cordance), alone and in combination, in predicting antidepressant response at week 12 of pharmacotherapy treatment using ML. In this study, comprised of a balanced sample of eventual antidepressant treatment responders/non-responders, we focused on Random Forest, though six other ML approaches were compared (such comparisons are currently lacking). To our knowledge, this is the first known study assessing the predictive utility of source-localized EEG current density across brain regions using ML. Further, in addition to sex and age, *individual* depression symptom questionnaire item scores were assessed in predicting antidepressant response (alone/in combination with EEG data). Most comparable work generally includes only *total* scores. This work expands on the ever-growing body of research investigating the utility of ML tools in aiding with antidepressant response prediction, with the broader aim of improving clinical care by integrating precision-based and personalized interventions in treating MDD.

Briefly, when considering each dataset separately, we found 50 eLORETA features to be predictive of response. Predictive delta eLORETA features were largely baseline and right-localized; those of theta were mainly baseline occipital and week 1 left temporo-parietal. Predictive eLORETA alpha_1_ features were mainly week 1 and left temporally-localized, while predictive alpha_2_ features were baseline and localized to the left parahippocampal and right pre/frontal cortex. Predictive eLORETA beta features were localized to the precentral gyrus and posterior regions at week 1. Overall, fewest predictive eLORETA features exited for delta, and most for alpha_1_/theta. Regarding scalp EEG power, 88 features were predictive. Predictive EEG delta features were largely week 1 and right-localized; those of EEG theta were generally baseline frontal and occipital, while week 1 were diffuse (not occipital). EEG alpha_1_ predictive features were generally baseline and frontal, while those of alpha_2_ were diffuse at baseline, and parieto-occipital at week 1. Diffuse predictive EEG beta features existed at both timepoints. Theta and alpha_2_ were the most predictive scalp EEG features. Clinical and demographic data consisted of 31 predictive features; the most salient being “concentration difficulty” score at week 1, followed by “sadness” score and total MADRS score changes from baseline to week 1. When all of the features were included into one ML experiment, the predictive utility of this model was high (PPV: 0.99; NPV: 0.81). When the *most* important features were identified in the final experiment, 12 predictive features were extracted, with the most predictive being baseline scalp EEG theta at Fp_2_, followed by baseline scalp EEG alpha_2_ at P_8_, and baseline scalp EEG alpha_1_ at Fp_2_. We found that a model combining all important features (**Experiment D**) had very high specificity (0.99), i.e., true negative rate, with a modest sensitivity (0.77), i.e., true positive rate, and a classification accuracy of 0.88. The high PPV indicates that the model is able to predict, with a high degree of certainty, that a given patient will truly be an eventual responder by week 12. The model which contains the *most* important features (**Experiment E**) had similar accuracy measures, however, the sensitivity, NPV, and overall accuracy were lower than those in **Experiment D**. However, this can be explained by the feature extraction process (we are reducing the number of features from 171 to 12). These data support the utility of EEG biomarkers in antidepressant response prediction.

The majority of studies using non-ML approaches assessing the predictive utility of EEG data have focused on midline, generally rostral anterior cingulate cortex (ACC)-localized theta current density. The rACC is known to be a region critical in conflict resolution as well as in coordinating the physiological response to conflict. Several studies have shown that higher pre-treatment rACC-theta tends to be associated with a favorable antidepressant response (78–81), though notable exceptions exist (82, 83). There is also work suggesting that early changes in rACC-theta may be associated with antidepressant response (49). Further, pre-treatment rACC theta has been shown to be predictive of placebo response. As such, activity in this region and frequency band may be reflective of “response readiness” (or malleability within a region highly implicated in MDD) rather than solely physiological changes induced by antidepressant drugs (84). In fact, the possibility of a placebo response driving some of the findings reported herein cannot be discounted. Interestingly, one group noted that rACC-delta was predictive of response (85), though a high correlation existed between delta and theta current density. Thus, although the rACC appears to be an important nexus in antidepressant response prediction, is seems worthwhile to investigate current density across all EEG bands and brain regions in relation to response (as was done in the current study), in order to identify other potentially predictive features of response.

In the eLORETA dataset, we found many features -across all bands, in diffuse brain areas, and at both timepoints- to be associated with response. Further, in this study, rACC-theta was not a predictive feature of response, though baseline alpha_2_ current density in this region was. Overall, the response predictive regions that alpha_2_ current density was localized to are regions typically associated with structural and functional alterations in MDD, such as the subgenual ACC [sgACC; (81)], parahippocampal regions (86) and pre/frontal regions (87). In the final model of most relevant features, only baseline sgACC-alpha_2_, week 1 alpha_1_ in the left middle temporal gyrus and alpha_1_/theta localized to the left auditory cortex contributed to response prediction. This is in keeping with the importance of the sgACC in response prediction (though not necessarily theta-localized), while the implication of the auditory cortex may be related to its high innervation by serotonergic fibers (88), though, this interpretation is speculative.

When considering the literature on the utility of resting-state scalp EEG in predicting response using non-ML approaches, the literature -while extensive- is rather inconsistent. In general, pre-treatment alpha power has been shown to differentiate responders and non-responders (49, 78, 89–92). However, in a large sample of depressed patients, the International Study to Predict Optimized Treatment in Depression (iSPOT-D) did not observe this [frontal alpha asymmetry was predictive of response in females; (82)]. Similarly, there has been an association between treatment response and baseline scalp EEG theta activity. Several groups reported that increased frontal/diffuse scalp-level theta was associated with antidepressant non-response (92–94) while others noted the opposite (95, 96) (i.e., increased fronto-midline theta was associated with a favorable outcome). As outlined in the introduction, several studies exist with respect to the predictive abilities of prefrontal theta cordance, wherein decreases in prefrontal theta cordance early in the course of antidepressant treatment tend to be associated with treatment outcome (32–34). Further, combining theta cordance data with clinical scores strengthened response predication (97). Such work underscores the importance of diverse data in improving predictive algorithms.

Assessments of the scalp-level EEG dataset revealed a degree of similarly between the scalp-level and eLORETA predictive features; though the overlap was far from perfect. Further, in the final predictive experiment of most relevant features, more scalp-level EEG features were predictive of response. In the current study, ML indicated that alpha was one of the most predictive bands of antidepressant response outcome, replicating previous work (49, 78, 89–92). Indeed, in the final model, alpha_1/2_ power at frontopolar electrodes and alpha/delta/theta power at P_8_ were most predictive scalp-level EEG features. The predictive utility of frontopolar electrodes fits with the findings of Al-Kaysi et al. (43), who found that frontal sites were most predictive of depression symptom and cognitive improvement (vs. non-improvement) following tDCS using ML approaches (i.e., SVM, linear discriminate analysis, extreme learning machine). The importance of frontopolar electrodes is also in keeping with the work on frontal theta cordance, which focuses on pre/frontal sites. The importance of the P_8_ site fits with the work associating the parietal region with anxious arousal (76, 98, 99). Most individuals with depression exhibit heighted anxiety, either at sub-clinical or clinical levels (a handful of our participants had co-morbid anxiety, and many had sub-clinical anxiety features). Finally, alterations in fronto-parietal networks are implicated in MDD (100), thus, predictive scalp-level EEG features at these sites may reflect this. Indeed, future work should investigate the utility of EEG connectivity (particularly between fronto-parietal regions) which may also have predictive value, as shown by others (41, 42).

Early depression symptom changes have been associated with eventual response. A meta-analysis found that a 20% reduction on the Hamilton Rating Scale for Depression (HAMD-17) within 2 weeks post-treatment initiation was predictive of later response and remission with a high sensitivity [81–87%; (101)]. In another meta-analysis, Wagner et al. (102) found that early improvement (>20/25% HAMD/MADRS reductions from baseline to week 1 or 2) predicted later response with high sensitivity (85%), but lower specificity (54%). However, analyses of individual trajectories of symptom change found that both early and delayed improvement are equally common (51% showed a delayed response); thus, eventual response cannot be predicted from early assessments in all patients (103). In terms of specific or individual symptom score changes being predictive of response, one study found that individual symptoms performed better than total improvement scores, though the difference was small (104). Such findings suggest that both total depression symptom scores or individual items cannot be solely relied upon as a predictive tool of response; thus, combining clinical measures with EEG may yield higher predictive accuracy. Interestingly, we found that “concentration difficulty” scores at week 1, as well as “sadness” and total MADRS score changes were most predictive; the “sadness” change scores and “concentration difficulty” scores at week 1 were also included in the most predictive/final feature model. Concentration difficulties and general cognitive dysfunction are potential risk factors for MDD relapse, as well as being associated with psychosocial and impaired daily functioning in the disorder (105). As such, the importance of concentration difficulties in relation to response prediction is noteworthy.

Early prediction of a negative response/non-response is just as important as positive response prediction. For instance, if one could predict -with a high degree of certainty- that a given patient will not respond to a particular treatment shortly after treatment initiation, then an adjunctive or alternative treatment could be offered. Indeed, there are EEG-based ML initiatives that attempt to do just that: characterize the probability of response and non-response to antidepressant interventions, and provide treatment recommendations. For instance, PEER (Psychiatric Electroencephalography Evaluation Registry) is a registry which selects an individual's medication class, independent of diagnosis, based on a pre-treatment EEG indices (the PEER database consists of EEG data from thousands of patients and associated clinical outcomes). The PEER report provides the probability of both response and non-response to medication classes. Preliminary data from PEER trials show promise [e.g., (106)].

There is limited published data to which we can directly compare our findings. Khodayari-Rostamabad et al. (39), who carried out ML using EEG to predict antidepressant treatment outcome at 2-weeks post-treatment initiation, found similar prediction accuracy [though the ML techniques were different and they did not report F1-scores; further, their sample was smaller (*N* = 22)]. However, different features were selected in the two studies, as we did not include coherence measures, which were found to be the most predictive features by Khodayari-Rostamabad et al. Another group that used ML techniques for predicting antidepressant response following rTMS found that elevated theta connectivity (particularly frontal to posterior connectivity) at baseline and week 1 was most predictive, with relatively high sensitivity (0.84) and specificity (0.89). Al-Kaysi et al. (43) used ML approaches to predict which MDD patients would respond to tDCS-induced cognitive and depressive symptom changes (MADRS total score) found that frontal electrodes were most useful (and that fronto-central connectivity was highly predictive). Despite a small sample size, they were able to correctly classify a substantial proportion of their patients correctly with respect to response (frequency bands were considered together).

## Future Directions and Limitations

While existing research, coupled with data from the current study, is difficult to synthesize, certain themes exist. First, the most predictive emergent features of antidepressant response using ML (and non-ML) approaches tend to be either from alpha and/or theta EEG bands. While features from other bands are also valuable, discarding them seems to be helpful in tackling the problem of dimensionality and building generalizable predictive models, and therefore improving response prediction. Thus, focusing on alpha/theta bands may be reasonable. Second, pre/frontal regions tend to be most associated with response, though the contribution of parietal regions is also notable. Third, combining various EEG measures (e.g., connectivity, coherence, power) may yield the most powerful ML predictive models. A recent ML study, which employed a wavelet-based technique for predicting response to SSRI treatment in MDD, found a classification accuracy of 87.5% using pre-treatment wavelet data in delta and theta frequencies in frontal and temporal regions (107). Thus, this method may be another useful contributor of response prediction in comparable future ML work. However, the utility of these measures has to be balanced with practical considerations. For example, the time associated with extracting eLORETA current density is substantial; further, localization of sources using eLORETA is based on several assumptions-when these are violated, source-localization can be flawed. While we found that eLORETA features added predictive value, the contribution of scalp-level EEG power was greater. Moving forward, if ML approaches using EEG data are to be viable tools for antidepressant response prediction, input features should require limited data clean-up and pre-processing. As such, source-localized EEG features (as well as wavelet analyses) may be less practical when considering clinical applications. Our selection of frequency band cut-offs (though not atypical) may also vary from published work (the same is true for our filter parameters). Specifically, with respect to the beta band, we analyzed frequencies ranging for 13–30 Hz (with a 0.1–30 Hz bandpass filter); as such, the influence of upper beta values (i.e., closer to 30 Hz) may have been attenuated. This, in turn, could have altered the prediction results from the beta band. Similarly, the predictive utility of gamma was not considered. Another methodological consideration is reference choice [e.g., average, linked-mastoid, reference electrode standardization technique [REST]; (108)]. In the current study, linked-mastoids were used as the reference. However, we acknowledge that this may introduce a degree of physiological noise that may have altered the data and thus classification accuracies. Future ML studies may benefit from constructing datasets with different reference montages, and comparing accuracy. This would also aid in potentially standardizing reference choice in the context of ML. In a similar vein, there is a need for combining datasets from a large number of centers/different groups to ensure that ML-identified response prediction features are properly tested on independent cases (i.e., models built on one large dataset but tested on another large dataset). Further, large datasets are required to extract features predictive of response to various antidepressant medication classes, which is critical information for personalized care. Such initiatives are already in place, and the results of such efforts are eagerly awaited. A final point is that the measures included in the current study were based on commonly-employed EEG features (i.e., power, source-localized activity, cordance). However, this approach may be obscuring potentially useful information that we did not think to include. Therefore, future studies may be optimized by employing a more data-driven process of feature extraction (though, there are practical considerations that must be considered).

It is also worth commenting on the value of including clinical features in predictive ML algorithms using EEG data. In the current work, we found that both individual item scores and total scores were predictive of response. Further, it seems that cognitive symptom scores, such as concentration, may be particularly important. Interestingly, in the current study, age and sex did not have as high a predictive value; however, given that this information is not difficult to obtain, it should be included in future predictive ML work (but, is generally not). EEG features at both baseline and week 1 were predictive of response. Thus, if possible, data from before and shortly after treatment commencement should be included in prediction algorithms (though, from a practical point of view, this may not always be possible). We also did not include changes in theta cordance from baseline to week 1, which has been more consistently utilized in differentiating antidepressant responders/non-responders; this may have contributed to improving our final predictive models. Finally, while our prediction accuracy scores for each ML method were derived from ROC curves, it has been suggested that meaningful qualitative conclusions should be drawn from ROC analyses that include >100 cases. This is especially important when differences between categories are subtle (109). As such, in the context of the current and future work, a larger sample size would be optimal for drawing values from ROC curves. In other words, larger samples (*N* > 100 participants) would ostensibly yield more valid sensitivity and specificity values.

There are several considerations that warrant discussion from a ML perspective. First, there is no hard-and-fast rule to determine a specific sample size for building a stable predictive model. Traditionally, this is based on the trade-off between factors such as feature (dimension) space, sample size, distribution of samples across classes, the nature of the data, and whether the problem is a binary or a complex multi-classification problem. Hence, while a larger sample size is always preferable, and is certainly recommended for comparable future work (i.e. multi-center, multi-group data), our predictive models should be considered appropriate in this context for the following reasons: First, the ML approaches we used were based on a simple binary classification problem, and the number of features was generally less than the sample size (which aids with the issue of overfitting; see Supplementary Information). Further, the target feature was balanced in terms of the number of responders/non-responders. Finally, we also attempted to avoid overfitting by applying cross-validation and regularization methods.

Additionally, although there are positive aspects to using Random Forests, which was our primary ML focus (e.g., easy to apply), there are caveats which must be considered. Namely, features can be correlated, and any of these correlated features can be used as a predictor in the model. As a result, once a predictive feature is selected, the importance of other correlated features decreases, which means that even strong features can be ranked with a lower importance. While this reduces overfitting, it may lead to the erroneous assumption that the certain predictors are significantly less important (65). We attempted to deal with this by including various other ML approaches in the current paper. Generally, if a specific set of features was ranked as having high predictive utility (e.g., alpha/theta scalp-level EEG) with RF, it tended to be highly predictive using the other approaches.

## Conclusion

In conclusion, a set of predictive methods in ML applied to our resting-state EEG dataset proved to be a viable approach for extracting salient predictive features of antidepressant treatment efficacy in patients with MDD. Importantly, the combination of datasets seems to provide enhanced predictive ability. A recent meta-analysis found that, as of now, quantitative EEG does not appear to be clinically reliable in the prediction of antidepressant treatment response; one suggested explanation is that depression itself is heterogeneous, therefore, the prediction of response via EEG may be clouded by differences in patient sub-groups or characteristics (110). This supports the combination of both electrophysiological as well as individual depression symptoms to improve predictive ability and future reliability. However, the generalizability of the current findings needs to be assessed in larger populations, and with different pharmacological antidepressant agents and/or other forms of antidepressant interventions. While it is premature to conclude whether this EEG-based technology will be suitable for integration in daily clinical practice, our data, along with those of others, suggests that the use of ML approaches with scalp-level EEG, clinical/demographic features, and EEG source-localization, may have significant potential in defining optimal predictors which can be used to guide and personalize antidepressant treatment.

## Data Availability Statement

The datasets for this manuscript are not publicly available because, in the original study, patients did not consent to public sharing of their data. However, anonymized data can be accessed upon request, by emailing Dr. Natalia Jaworska (Natalia.Jaworska@theroyal.ca).

## Ethics Statement

All subjects gave written informed consent in accordance with the Declaration of Helsinki. The protocol was approved by the Royal Ottawa Health Care Group and University of Ottawa Social Sciences and Humanities Research Ethics Boards.

## Author Contributions

NJ recruited and tested the patients, processed the EEG files, and wrote the manuscript. SdlS processed and assembled EEG and clinical data files and assisted in writing and editing the manuscript. M-HI was responsible for performing ML experiments, writing the ML methods section, and editing the manuscript. PB the clinical lead on the project, screened, interviewed, and diagnosed all patients and helped to edit the paper. VK was responsible for developing the research questions and overseeing the project as well as editing the manuscript.

### Conflict of Interest Statement

The authors declare that the research was conducted in the absence of any commercial or financial relationships that could be construed as a potential conflict of interest.

## References

[1] Collins PY Patel V Joestl SS March D Insel TR Daar AS. Grand challenges in global mental health. Nature (2011) 475:27–30. 10.1038/475027a 21734685 PMC3173804

[2] Druss BG Schlesinger M Allen HM. Depressive symptoms satisfaction with health care and 2-year outcomes in an employed population. Am J Psychiat. (2001) 158:731–4. 10.1176/appi.ajp.158.5.731 11329394

[3] Evans DL Charney DS. Mood disorders and medical illness: a major public health problem. Biol Psychiatry (2003) 54:177–80. 10.1016/S0006-3223(03)00639-5 12893090

[4] Moussavi S Chatterji S Verdes E Tandon A Patel V Ustun B. Depression, chronic diseases, and decrements in health:results from the World Health Surveys. Lancet (2007) 370:851–8. 10.1016/S0140-6736(07)61415-9 17826170

[5] Frasure-Smith N Lespérance F Gravel G Masson A Juneau M Talajic M. Social support, depression, and mortality during the first year after myocardial infarction. Circulation (2000) 101:1919–24. 10.1161/01.CIR.101.16.1919 10779457

[6] Pigott HE Leventhal AM Alter GS Boren JJ. Efficacy and effectiveness of antidepressants: current status of research. Psychother Psychosom. (2010) 79:267–79. 10.1159/000318293 20616621

[7] Trivedi MH Rush AJ Wisniewski SR Nierenberg AA Warden D Ritz L. Evaluation of outcomes with citalopram for depression using measurement-based core in STAR^*^D:implications for clinical practice. Am J Psychiatry (2006) 163:28–40. 10.1176/appi.ajp.163.1.28 16390886

[8] Malone DC. A budget-impact and cost-effectiveness model for second-line treatment of major depression. J Manag Care Pharm. (2007) 13:S8–18. 10.18553/jmcp.2007.13.s6-a.8 17874482 PMC10437458

[9] Rush AJ Trivedi MH Wisniewski SR Nierenberg AA Stewart JW Warden D. Acute and longer-term outcomes in depressed outpatients requiring one or several treatment step:a STAR^*^D report. Am J Psychiatry (2006) 163:1905–17. 10.1176/ajp.2006.163.11.1905 17074942

[10] Wagner JA. Overview of biomarkers and surrogate endpoints in drug development. Dis Markers (2002) 18:41–6. 10.1155/2002/929274 12364809 PMC3851644

[11] Labermaier C Masana M Muller M. Biomarkers predicting antidepressant treatment response:how can we advance the field? Dis Markers (2013) 35:23–31. 10.1155/2013/984845 24167346 PMC3774965

[12] Leuchter AF Cook IA Hamilton SP Narr KL Toga A Hunter AM. Biomarkers to predict antidepressant response. Curr Psychiat Rep. (2010) 12:553–62. 10.1007/s11920-010-0160-4 20963521 PMC2965366

[13] Insel T Cuthbert B Garvey M Heinssen R Pine DS Quinn K Research Domain Criteria (RDOC):Toward a new classification framework for research or mental disorders. Am J Psychiatry (2010) 167:748–51. 10.1176/appi.ajp.2010.09091379 20595427

[14] de Almeida CJR Phillips ML Distinguishing between unipolar depression and bipolar depression:Current and future clinical and neuroimaging perspectives. Biol Psychiatry (2013) 73:111–8. 10.1016/j.biopsych.2012.06.010 22784485 PMC3494754

[15] Hasler G Northoff G. Discovering imaging endophenotypes for major depression. Mol Psychiatry. (2011) 16:604–19. 10.1038/mp.2011.23 21602829

[16] Hasler G Drevets WC Manji HK Charney DS. Discovering endophenotypes for major depression. Neuropsychopharmacology (2004) 29:1765–81. 10.1038/sj.npp.1300506 15213704

[17] McGrath CL Kelley ME Holtzheimer PE Dunlop BW Craighead WE Franco AR. Toward a neuroimaging treatment selection biomarker for major depressive disorder. JAMA Psychiatry (2013) 70:821–9. 10.1001/jamapsychiatry.2013.143 23760393 PMC4413467

[18] Niciu MJ Mathews DC Nugent AC Ionescu DF Furey ML Richards EM. Developing biomarkers in mood disorders research through the use of rapid-acting antidepressants. Depress Anxiety (2014) 31:297–307. 10.1002/da.22224 24353110 PMC3984598

[19] Schmidt HD Shelton RC Duman RS. Functional biomarkers of depression:Diagnosis, treatment and pathophysiology. Neuropsychopharmacology (2011) 36:2375–94. 10.1038/npp.2011.151 21814182 PMC3194084

[20] Schneider B Prvulovic D. Novel biomarkers in depression. Curr Opin Psychiatry (2013) 26:47–53. 10.1097/YCO.0b013e32835a5947 23154643

[21] Wise T Cleare AJ Herane A Young AH Arnone D. Diagnostic and therapeutic utility of neuroimaging in depression:an overview. Neuropsychiatr Dis Treat. (2014) 10:1509–22. 10.2147/NDT.S50156 25187715 PMC4149389

[22] Ritsner M Gottesman E Chapter 1: Where do we stand in the quest for neuropsychiatric biomarkers and what next? In: Ritsne MS editor. The Handbook of Neuropsychiatric Biomarkers, Endophenotypes and Genes, New York, NY: Springer (2009), p. 3–17. 10.1007/978-1-4020-9464-4_1

[23] Michel C Murray M Towards the utilization of EEG as a brain imaging trial. Neuroimage (2012) 61:371–85. 10.1016/j.neuroimage.2011.12.039 22227136

[24] Alhaj H Wisniewski G McAllister-Williams RH. The use of EEG in measuring therapeutic drug action: focus on depression and antidepressants. J Psychopharmacol (2011) 25:1175–91. 10.1177/0269881110388323 21106608

[25] Knott V Mahoney C Kennedy S Evans K. EEG correlates of acute and chronic paroxetine treatment in depression. J Affect Dis. (2002) 69:241–9. 10.1016/S0165-0327(01)00308-1 12103473

[26] Baskaran A Milev R McIntyre RS. The neurobiology of the EEG biomarker as a predictor of treatment response in depression. Neuropharmacology (2012) 63:507–13. 10.1016/j.neuropharm.2012.04.021 22569197

[27] Iosifescu DV. Electroencephalography-derived biomarkers of antidepressant response. Harv Rev Psychiatry (2011) 19:144–54. 10.3109/10673229.2011.586549 21631160

[28] Leuchter AF Cook IA Hunter A Korb A. Use of clinical neurophysiology for the selection of medication in the treatment of major depressive disorder: the state of the evidence. Clin EEG Neurosci. (2009) 40:78–83. 10.1177/155005940904000207 19534301

[29] Olbrich S Arns M. EEG biomarkers in major depressive disorder:Discriminative power and prediction of treatment response. Int Rev Psychiatry (2013) 25:604–18. 10.3109/09540261.2013.816269 24151805

[30] Neuper C Pfurtscheller G. Event-related dynamics of cortical rhythms:frequency-specific features and functional correlates. Int J Psychophysiol. (2001) 43:41–58. 10.1016/S0167-8760(01)00178-7 11742684

[31] Leuchter AF Cook IA Lufkin RB Dunkin J Newton TF Cummings JL. Cordance:a new method for assessment of cerebral perfusion and metabolism using quantitative electroencephalography. Neuroimage (1994) 1:208–19. 10.1006/nimg.1994.1006 9343572

[32] Cook IA Leuchter AF Witte E Abrams M Uijtdehaage SH Stubbeman W. Neurophysiologic predictors of treatment response to fluoxetine in major depression. Psychiatry Res. (1999) 85:263–73. 10.1016/S0165-1781(99)00010-4 10333379

[33] Bares M Brunovsky M Kopecek M Novak T Stopkova P Kozeny J. Early reduction in prefrontal theta QEEG cordance value predicts response to venlafaxine treatment in patients with resistant depressive disorder. Eur Psychiatry (2008) 23:350–5. 10.1016/j.eurpsy.2008.03.001 18450430

[34] Cook IA Leuchter AF Morgan M Witte E Stubbeman WF Abrams M. Early changes in prefrontal activity characterize clinical responders to antidepressants. Neuropsychopharmacology (2002) 27:120–31. 10.1016/S0893-133X(02)00294-4 12062912

[35] Lemm S Blankertz B Dickhaus T Müller KR. Introduction to machine learning for brain imaging. Neuroimage (2011) 56:387–99. 10.1016/j.neuroimage.2010.11.004 21172442

[36] Pereira F Mitchell T Botvinick M. Machine learning classifiers and fMRI:A tutorial overview. Neuroimage (2009) 45:5199–209. 10.1016/j.neuroimage.2008.11.007 19070668 PMC2892746

[37] Khodayari-Rostamabad A Reilly JP Hasey G Debruin H Maccrimmon D. Diagnosis of psychiatric disorders using EEG data and employing a statistical decision model. Conf Proc IEEE Eng Med Biol Soc. (2010) 2010:4006–9. 10.1109/IEMBS.2010.5627998 21097280

[38] Mohammadi M Al-Azab F Raahemi B Richards G Jaworska N Smith D. Data mining EEG signals in depression for their diagnostic value. BMC Med Inform Decis Mak. (2015) 15:108. 10.1186/s12911-015-0227-6 26699540 PMC4690290

[39] Khodayari-Rostamabad A Reilly JP Hasey GM de Bruin H Maccrimmon DJ. A machine learning approach using EEG data to predict response to SSRI treatment for major depressive disorder. Clin Neurophysiol. (2013) 124:1975–85. 10.1016/j.clinph.2013.04.010 23684127

[40] Rabinoff M Kitchen CM Cook IA Leuchter AF. Evaluation of quantitative EEG by classification and regression trees to characterize responders to antidepressant and placebo treatment. Opin Med Info J. (2011) 5:1–8. 10.2174/1874431101105010001 21603560 PMC3097432

[41] Bailey NW Hoy KE Rogasch NC Thomson RH McQueen S Elliot D. Differentiating responders and non-responders to rTMS treatment for depression after one week using resting EEG connectivity measures. J Affect Disord. (2019) 242:68–79. 10.1016/j.jad.2018.08.058 30172227

[42] Bailey NW Hoy KE Rogasch NC Thomson RH McQueen S Elliot D. Responders to rTMS for depression show increased fronto-midline theta and theta connectivity compared to non-responders. Brain Stimul. (2018) 11:190–203. 10.1016/j.brs.2017.10.015 29128490

[43] Al-Kaysi AM Al-Ani A Loo CK Breakspear M Boonstra TW. Predicting brain stimulation treatment outcomes of depressed patients through the classification of EEG oscillations. Conf Proc IEEE Eng Med Biol Soc. (2016) 2016:5266–9. 10.1109/EMBC.2016.7591915 28269452

[44] Crown WH. Potential application of machine learning in health outcomes research and some statistical cautions. Value Health (2015) 18:137–40. 10.1016/j.jval.2014.12.005 25773546

[45] Pascual-Marqui RD Pascual-Montano AD Lehmann D Exact low resolution brain electromagnetic tomography (eLORETA). Neuroimage (2006) 31:S86.

[46] Montgomery SA Asberg M. A new depression scale designed to be sensitive to change. Br J Psychiatry (1979) 134:382–9. 10.1192/bjp.134.4.382 444788

[47] Howland RH Wilson MG Kornstein SG Clayton AH Trivedi MH Wohlreich MM. Factors predicting reduced antidepressant response:experience with the SNRI duloxetine in patients with major depression. Ann Clin Psychiatry (2008) 20:209–18. 10.1080/10401230802437639 19034753

[48] Stewart JW McGrath PJ Blondeau C Deliyannides DA Hellerstein D Norris S. Combination antidepressant therapy for major depressive disorder: speed and probability of remission. J Psychiatr Res. (2014) 52:7–14. 10.1016/j.jpsychires.2013.12.001 24485847

[49] Jaworska N Blondeau C Tessier P Norris S Fusee W Blier P. Examining relations between alpha power as well as anterior cingulate cortex-localized theta activity and response to single or dual antidepressant pharmacotherapies. J Psychopharmacol (2014) 28:587–95. 10.1177/0269881114523862 24557661

[50] First M Gibbon M Spitzer R Structured Clinical Interview for DSM-IV Axis II Personality Disorder (SCID-II). Washington, DC: American Psychiatric Press Inc (1997).

[51] Gratton G Coles MG Donchin E. A new method for off-line removal of ocular artifact. Electroencephalogr Clin Neurophysiol. (1983) 55:468–84. 10.1016/0013-4694(83)90135-9 6187540

[52] Phillips C Rugg M D Fristont KJ. Systematic regularization of linear inverse solutions of the EEG source localization problem. Neuroimage (2002) 17:287e301. 10.1006/nimg.2002.1175 12482084

[53] Mulert C Jäger L Propp S Karch S Störmann S Pogarell O. Sound level dependence of the primary auditory cortex:simultaneous measurement with 61-channel EEG and fMRI. Neuroimage (2005) 28:49e58. 10.1016/j.neuroimage.2005.05.041 16006148

[54] Pizzagalli D Oakes T Fox A Chung MK Larson CL Abercrombie HC. Functional but not structural subgenual prefrontal cortex abnormalities in melancholia. Mol Psychiatry (2004) 9:393e405. 10.1038/sj.mp.4001469 14699431

[55] Leuchter AF Uijtdehaage SH Cook IA O'Hara R Mandelkern M. Relationship between brain electrical activity and cortical perfusion in normal subjects. Psychiatry Res. (1999) 90:125–40. 10.1016/S0925-4927(99)00006-2 10482384

[56] Grus J Data Science from Scratch:First Principles with Python. Sebastopol, CA: O'Reilly Media (2015).

[57] Han J Pei J Kamber M Data Mining:Concepts and Techniques. Waltham, MA: Elsevier (2011).

[58] Juszczak P Tax D Duin RPW Feature scaling in support vector data description. In: Proceedings of ASCI. Citeseer (2002). p. 95–102.

[59] Geurts P Damien E Louis W Extremely randomized trees. Mach Learn. (2006) 63:3–42. 10.1007/s10994-006-6226-1

[60] Lerman R Yitzhaki S A note on the calculation and interpretation of the Gini index. Econ Lett. (1984) 15:363–8. 10.1016/0165-1765(84)90126-5

[61] Heiko H Kernel PCA for novelty detection. Pattern Recogn. (2007) 40:863–74. 10.1016/j.patcog.2006.07.009

[62] Schölkopf B Smola A Müller K-R Nonlinear component analysis as a kernel eigenvalue problem. Neural Comput. (1998) 10:1299–319. 10.1162/089976698300017467

[63] Sidhu GS Asgarian N Greiner R Brown MR. Kernel principal component analysis for dimensionality reduction in fMRI-based diagnosis of ADHD. Front Syst Neurosci. (2012) 6:74. 10.3389/fnsys.2012.00074 23162439 PMC3494168

[64] Breiman L Random forests. Mach Learn. (2001) 45:5–32. 10.1023/A:1010933404324

[65] Strobl C Boulesteix AL Zeileis A Hothorn T. Bias in random forest variable importance measures:illustrations, sources and a solution. BMC Bioinformatics (2007) 8:25. 10.1186/1471-2105-8-25 17254353 PMC1796903

[66] Probst P Boulesteix A-L To tune or not to tune the number of trees in random forest. J Mach Learn Res. (2018) 18:1–18.

[67] Oshiro TM Perez PS Baranauskas JA How many trees in a random forest? Machine. In: Learning and Data Mining in Pattern Recognition. Berlin: Springer (2012). p. 154–168. 10.1007/978-3-642-31537-4_13

[68] Breiman L Classification and Regression Trees. New York, NY: Routledge (2017). 10.1201/9781315139470

[69] Loh W-Y Classification and regression trees. Wiley Int Rev. (2011) 1:14–23. 10.1002/widm.8

[70] Cortes C Vapnik V Support-vector networks. Mach Learn. (1995) 20:273–97. 10.1007/BF00994018

[71] Freund Y Schapire R A decision-theoretic generalization of on-line learning and an application to boosting. J Comp Syst Sci. (1997) 55:119–39. 10.1006/jcss.1997.1504

[72] John Lu QZ The elements of statistical learning:data mining, inference, and prediction. J R Stat Soc Ser A. (2010) 173:693–4. 10.1111/j.1467-985X.2010.00646_6.x

[73] Mitchell TM Logistic regression. Mach Learn. (2005). 10:701.

[74] Zweig MH Campbell G. Receiver-operating characteristic (ROC) plots:a fundamental evaluation tool in clinical medicine. Clin Chem. (1993) 39:561–77. 8472349

[75] Powers DM Evaluation:from precision, recall and F-measure to ROC, informedness, markedness and correlation. J Mach Learn Technol. (2011) 2:37–63.

[76] Myerson J Green L Warusawitharana M. Area under the curve as a measure of discounting. J Exp Anal Behav. (2001) 76:235–43. 10.1901/jeab.2001.76-235 11599641 PMC1284836

[77] Hunter AM Cook IA Greenwald SD Tran ML Miyamoto KN Leuchter AF. The antidepressant treatment response index and treatment outcomes in a placebo-controlled trial of fluoxetine. J Clin Neurophysiol. (2011) 28:478–82. 10.1097/WNP.0b013e318230da8a 21946361 PMC3188349

[78] Korb AS Hunter AM Cook IA Leuchter AF. Rostral anterior cingulate cortex theta current density and response to antidepressants and placebo in major depression. Clin Neurophysiol. (2009) 120:1313–9. 10.1016/j.clinph.2009.05.008 19539524 PMC2710394

[79] Mulert C Juckel G Brunnmeier M Karch S Leicht G Mergl R. Rostral anterior cingulate cortex activity in the theta band predicts response to antidepressive medication. Clin EEG Neurosci. (2007) 38:78–81. 10.1177/155005940703800209 17515172

[80] Narushima K McCormick LM Yamada T Thatcher RW Robinson RG. Subgenual cingulate theta activity predicts treatment response of repetitive transcranial magnetic stimulation in participants with vascular depression. J Neuropsychiatry Clin Neurosci. (2010) 22:75–84. 10.1176/jnp.2010.22.1.75 20160213 PMC3688059

[81] Pizzagalli D Pascual-Marqui RD Nitschke JB Oakes TR Larson CL Abercrombie HC. Anterior cingulate activity as a predictor of degree of treatment response in major depression:evidence from brain electrical tomography analysis. Am J Psychiatry (2001) 158:405–15. 10.1176/appi.ajp.158.3.405 11229981

[82] Arns M Bruder G Hegerl U Spooner C Palmer DM Etkin A. EEG alpha asymmetry as a gender-specific predictor of outcome to acute treatment with different antidepressant medications in the randomized iSPOT-D study. Clin Neurophysiol. (2016) 127:509–19. 10.1016/j.clinph.2015.05.032 26189209

[83] Konarski JZ Kennedy SH Segal ZV Lau MA Bieling PJ McIntyre RS. Predictors of nonresponse to cognitive behavioural therapy or venlafaxine using glucose metabolism in major depressive disorder. J Psychiatry Neurosci. (2009) 34:175–80. 19448846 PMC2674969

[84] Pizzagalli DA Webb CA Dillon DG Tenke CE Kayser J Goer F. Pretreatment rostral anterior cingulate cortex theta activity in relation to symptom improvement in depression: a randomized clinical trial. JAMA Psychiatry (2018) 75:547–54. 10.1001/jamapsychiatry.2018.0252 29641834 PMC6083825

[85] Rentzsch J Adli M Wiethoff K Gómez-Carrillo de Castro A Gallinat J. Pretreatment anterior cingulate activity predicts antidepressant treatment response in major depressive episodes. Eur Arch Psychiatry Clin Neurosis. (2014) 264:213–23. 10.1007/s00406-013-0424-1 23873091

[86] Vasic N Wolf ND Grön G Sosic-Vasic Z Connemann BJ Sambataro F. Baseline brain perfusion and brain structure in patients with major depression:a multimodal magnetic resonance imaging study. J Psychiatry Neurosci. (2015) 40:412–21. 10.1503/jpn.140246 26125119 PMC4622640

[87] Rigucci S Serafini G Pompili M Kotzalidis GD Tatarelli R. Anatomical and functional correlates in major depressive disorder:the contribution of neuroimaging studies. World J Biol Psychiatry (2010) 11:165–80. 10.3109/15622970903131571 19670087

[88] Hegerl U Gallinat J Juckel G. Event-related potentials. Do they reflect central serotonergic neurotransmission and do they predict clinical response to serotonin agonists? J Affect Disord. (2001) 62:93–100 10.1016/S0165-0327(00)00353-0 11172876

[89] Bruder GE Sedoruk JP Stewart JW McGrath PJ Quitkin FM Tenke CE. Electroencephalographic alpha measures predict therapeutic response to a selective serotonin reuptake inhibitor antidepressant:Pre- and post-treatment findings. Biol Psychiatry (2008) 63:1171–7. 10.1016/j.biopsych.2007.10.009 18061147 PMC2652474

[90] Knott VJ Telner JI Lapierre YD Browne M Horn ER. Quantitative EEG in the prediction of antidepressant response to imipramine. J Affect Disord. (1996) 39:175–84. 10.1016/0165-0327(96)00003-1 8856421

[91] Tenke CE Kayser J Manna CG Fekri S Kroppmann CJ Schaller JD. Current source density measures of electroencephalographic alpha predict antidepressant treatment response. Biol Psychiatry (2011) 70:388–94. 10.1016/j.biopsych.2011.02.016 21507383 PMC3142299

[92] Ulrich G Renfordt E Zeller G Frick K. Interrelation between changes in the EEG and psychopathology under pharmacotherapy for endogenous depression: a contribution to the predictor question. Pharmacopsychiatry (1984) 17:178–83. 10.1055/s-2007-1017433 6514780

[93] Arns M Drinkenburg WH Fitzgerald PB Kenemans JL. Neurophysiological predictors of non-response to rTMS in depression. Brain Stimul. (2012) 5:569–76. 10.1016/j.brs.2011.12.003 22410477

[94] Iosifescu DV Greenwald S Devlin P Mischoulon D Denninger JW Alpert JE. Frontal EEG predictors of treatment outcome in major depressive disorder. Eur Neuropsychopharmacol. (2009) 19:772–7. 10.1016/j.euroneuro.2009.06.001 19574030

[95] Arns M Etkin A Hegerl U Williams LM DeBattista C Palmer DM. Frontal and rostral anterior cingulate (rACC) theta EEG in depression:implications for treatment outcome? Eur Neuropsychopharmacol. (2015) 25:1190–200. 10.1016/j.euroneuro.2015.03.007 25936227

[96] Spronk D Arns M Barnett KJ Cooper NJ Gordon E. An investigation of EEG, genetic and cognitive markers of treatment response to antidepressant medication in patients with major depressive disorder:a pilot study. J Affect Disord. (2011) 128:41–8. 10.1016/j.jad.2010.06.021 20619899

[97] Bares M Novak T Kopecek M Brunovsky M Stopkova P Höschl C. The effectiveness of prefrontal theta cordance and early reduction of depressive symptoms in the prediction of antidepressant treatment outcome in patients with resistant depression: Analysis of naturalistic data. Eur Arch Psychiatry Clin Neurosci. (2015) 265:73–82. 10.1007/s00406-014-0506-8 24848366

[98] Heller W Nitschke JB Etienne MA Miller GA. Patterns of regional brain activity differentiate types of anxiety. J Abnorm Psychol. (1997) 106:376–85. 10.1037/0021-843X.106.3.376 9241939

[99] Manna CB Tenke CE Gates NA Kayser J Borod JC Stewart JW. EEG hemispheric asymmetries during cognitive tasks in depressed patients with high versus low trait anxiety. Clin EEG Neurosci. (2010) 41:196–202. 10.1177/155005941004100406 21077571 PMC3341096

[100] Kaiser RH Andrews-Hanna JR Wager TD Pizzagalli DA. Large-scale network dysfunction in major depressive disorder: a meta-analysis of resting-state functional connectivity. JAMA Psychiatry (2015) 72:603–11. 10.1001/jamapsychiatry.2015.0071 25785575 PMC4456260

[101] Szegedi A Jansen WT van Willigenburg AP van der Meulen E Stassen HH Thase ME. Early improvement in the first 2 weeks as a predictor of treatment outcome in patients with major depressive disorder:a meta-analysis including 6562 patients. J Clin Psychiatry (2009) 70:344–53. 10.4088/JCP.07m03780 19254516

[102] Wagner S Engel A Engelmann J Herzog D Dreimüller N Müller MB. Early improvement as a resilience signal predicting later remission to antidepressant treatment in patients with Major Depressive Disorder:Systematic review and meta-analysis. J Psychiatr Res. (2017) 94:96–106. 10.1016/j.jpsychires.2017.07.003 28697423

[103] Uher R Mors O Rietschel M Rajewska-Rager A Petrovic A Zobel A. Early and delayed onset of response to antidepressants in individual trajectories of change during treatment of major depression:a secondary analysis of data from the Genome-Based Therapeutic Drugs for Depression (GENDEP) study. J Clin Psychiatry (2011) 72:1478–84. 10.4088/JCP.10m06419 22127194

[104] de Vries Y Roest A Bos E Burgerhof JGM van Loo HM de Jonge P Predicting antidepressant response by monitoring early improvement of individual symptoms of depression: individual patient data meta-analysis. Br J Psychiatry (2018) 28:1–7. 10.1192/bjp.2018.122 PMC7557872 29952277

[105] Godard J Baruch P Grondin S Lafleur MF. Psychosocial and neurocognitive functioning in unipolar and bipolar depression: a 12-month prospective study. Psychiatry Res. (2012) 196:145–53. 10.1016/j.psychres.2011.09.013 22370154

[106] Iosifescu DV Neborsky RJ Valuck RJ. The use of the psychiatric electroencephalography evaluation registry (PEER) to personalize pharmacotherapy. Neuropsychiatr Dis Treat. (2016) 12:2131–42. 10.2147/NDT.S113712 27601908 PMC5003598

[107] Mumtaz W Xia L Mohd Yasin MA Azhar Ali SS Malik AS. A wavelet-based technique to predict treatment outcome for Major Depressive Disorder. PLoS ONE (2017) 12:e0171409. 10.1371/journal.pone.0171409 28152063 PMC5289714

[108] Yao D. A method to standardize a reference of scalp EEG recordings to a point at infinity. Physiol Meas. (2001) 22:693–711. 10.1088/0967-3334/22/4/305 11761077

[109] Metz CE. Basic principles of ROC analysis. Semin Nucl Med. (1978) 8:283–98. 10.1016/S0001-2998(78)80014-2 112681

[110] Widge AS Bilge MT Montana R Chang W Rodriguez CI Deckersbach T. Electroencephalographic biomarkers for treatment response prediction in major depressive illness: a meta-analysis. Am J Psychiatry (2018). [Epub ahead of print] 10.1176/appi.ajp.2018.17121358 30278789 PMC6312739

