# Supplementary material for: Leveraging Machine Learning Approaches for Predicting Antidepressant Treatment Response Using Electroencephalography (EEG) and Clinical Data
Source: Front Psychiatry. 2019 Jan 14;9:768. doi: 10.3389/fpsyt.2018.00768 (PMC6339954; doi:10.3389/fpsyt.2018.00768)
Supplement: Supplementary file 1 [file Data_Sheet_1.docx]

**SUPPLEMENTARY TABLES & FIGURES**

**Supplementary Figure 1.** Electrode montage for electroencephalographic (EEG) recordings (Brain Products, Gilching, Germany).

**
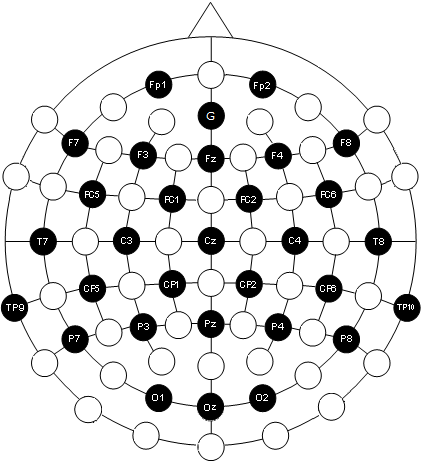
**

**Supplementary Table 1.** Summary of previous studies applying data mining techniques to the prediction of antidepressant treatment response using clinical electroencephalographic data

| **Study** | **ML technique** | **When?** | **Antidepressant Treatment** | **Prediction Accuracy** |
| --- | --- | --- | --- | --- |
| Rabinoff et al. (2011) | Classification & Regression Trees (CART) | Change from baseline (day 2, 7, 14, 28 or 56) | SSRI, SNRI | Sensitivity: 0.85  Specificity: 1  Accuracy: 0.93 |
| Khodayari-Rostamabad et al. (2013) | Feature selection, Classification using mixture of factor analysis (MFA) model, Predicted response = likelihood value  “Leave-n-out” randomized permutation cross-validation procedure | Pre-treatment | SSRI | Specificity: 80.9%  Sensitivity: 94.9%  Accuracy: 87.9% |
| Al-Kaysi et al. (2016) | Support vector machine  (SVM), linear discriminate analysis (LDA) & extreme learning machine (ELM) | Pre-treatment | tDCS | N/A |
| Mumtaz et al. (2017) | Feature matrix, dimension reduction, the logistic regression classifier  100 iterations of 10-fold cross-validation (10-CV) | Pre-treatment | SSRI | Sensitivity: 0.95  Specificity: 0.80  Accuracy: 0.88 |
| Bailey et al. (2018) | Multivariate machine learning model  (individual x feature) data matrix, with each feature normalized to zero mean and unit variance using a z-score; linear SVM classifier; 6-fold cross-validation | Pre-treatment & week 1 change | rTMS | Sensitivity: 0.90 Specificity: 0.92  Accuracy: 0.91 |
| Bailey et al. (2019) | Same as above | Pre-treatment & week 1 change | rTMS | Sensitivity: 0.84 Specificity: 0.89  Accuracy: 0.86 |

rTMS: repeated transcranial magnetic stimulation; SSRI: selective serotonin reupate inhibitor; SNRI: serotonin & norepinephrine reuptake inhibitor

**Supplementary Table 2.** Features predictive of antidepressant response from source-localized (eLORETA) activity across different electroencephalogram (EEG) bands at baseline and following one week of treatment. Within each band, features are organized based on importance (the most predictive feature listed first and least predictive listed last).

| **Band** | **Time/Session** | **Region** |
| --- | --- | --- |
| Delta | Baseline  Week 1  Baseline | Right lingual gyrus (BA17)  Left paracentral lobule (BA5)  Right middle frontal gyrus (BA6)  Right superior temporal gyrus (BA22)  Right inferior frontal gyrus (BA45)  Right cingulate gyrus (BA24) |
| Theta | Week 1  Baseline  Week 1  Baseline  Week 1 | Left transverse temporal gyrus (BA41)  Right lingual gyrus (BA17)  Left paracentral lobule (BA5)  Left superior temporal gyrus (BA42)  Right paracentral lobule (BA5)  Left lingual gyrus (BA17)  Left lingual gyrus (BA18)  Right posterior cingulate (BA29)  Left parahipoocampal gyrus (BA36)  Left parahipoocampal gyrus (BA35) |
| Alpha_1_ | Week 1  Baseline  Week 1 | Left transverse temporal gyrus (BA41)  Left superior temporal gyrus (BA42)  Right superior frontal gyrus (BA10)  Left parahippocampal gyrus (BA36)  Left fusiform gyrus (BA20)  Left precuneus (BA7)  Left middle temporal gyrus (BA21)  Left superior frontal gyrus (BA8)  Right anterior cingulate (BA33)  Left paracentral lobule (BA5)  Left parahippocampal gyrus (BA34)  Left transverse temporal gyrus (BA43)  Left parahippocampal gyrus (BA35) |
| Alpha_2_ | Baseline  Week 1  Baseline | Right subcallosal gyrus (BA25)  Right anterior cingulate (BA32)  Right inferior frontal gyrus (BA47)  Left parahippocampal gyrus (BA34)  Left parahippocampal gyrus (BA28)  Right middle frontal gyrus (BA46)  Left transverse temporal gyrus (BA43)  Left posterior cingulate (BA23)  Right precentral gyrus (BA44) |
| Beta | Week 1  Baseline  Week 1  Baseline  Week 1  Baseline | Left precuneus (BA31)  Left posterior cingulate (BA30)  Left posterior cingulate (BA23)  Left precentral gyrus (BA4)  Right posterior cingulate (BA29)  Left posterior cingulate (BA29)  Right posterior cingulate (BA23)  Left precuneus (BA7)  Left middle frontal gyrus (BA9)  Left paracentral lobule (BA5)  Left postcentral gyrus (BA1)  Right cuneus (BA30)  Left cuneus (BA30) |

**Supplementary Figure 2.** Receiver operator curve (ROC) & area under the curve (AUC) scores for each of the source-localized EEG bands (eLORETA-localized power density at delta [A], theta [B], alpha_1_ [C], alpha_2_ [D], beta [E]) using Random Forest.

**
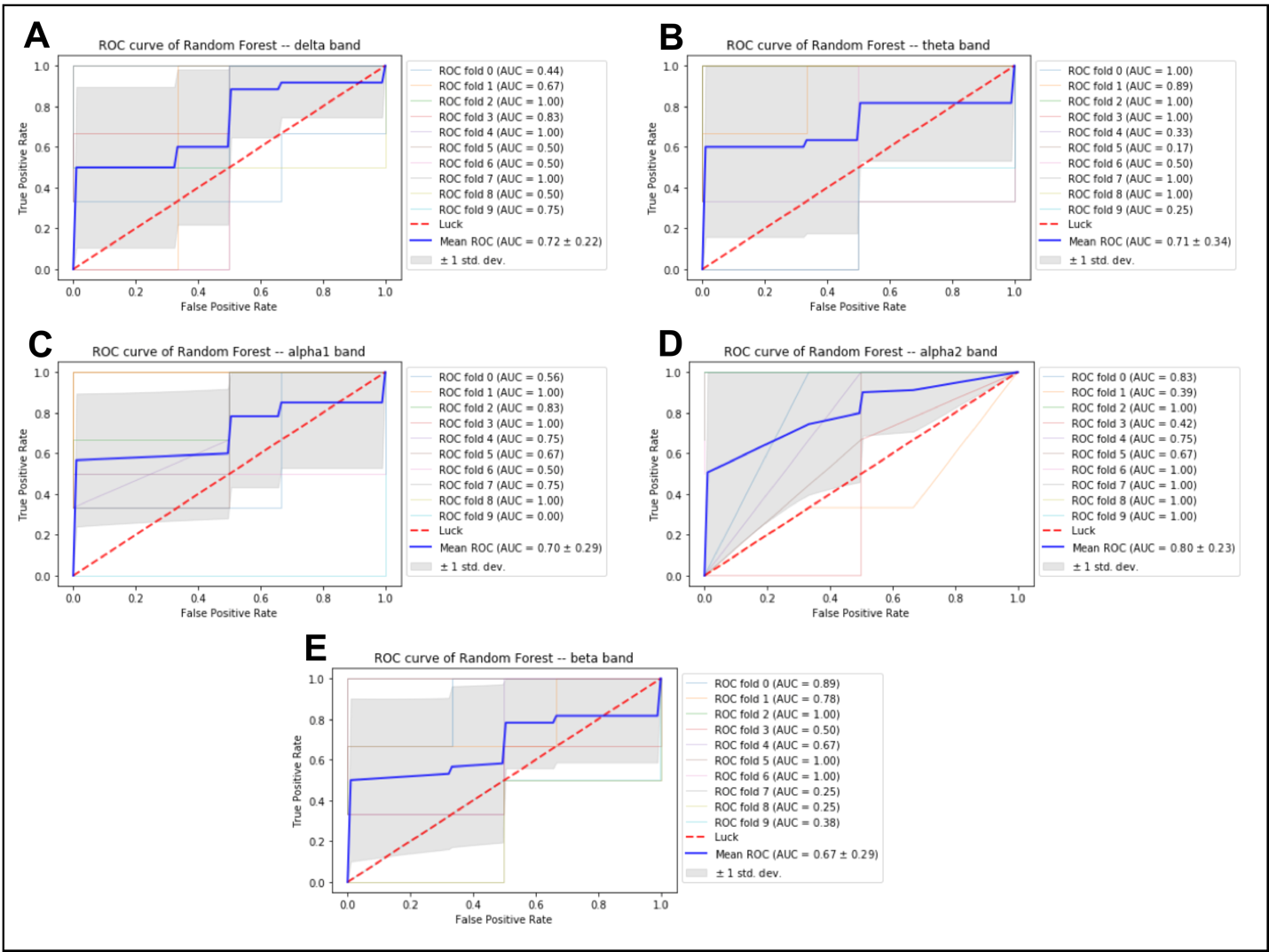
**

**Supplementary Table 3.** Features predictive of antidepressant response from surface-level power at different electroencephalogram (EEG) bands at baseline and following one week of treatment. Within each band, features are organized based on importance (the most predictive feature listed first and least predictive listed last).

| **Frequency Band** | **Time/Session** | **Electrodes/Features** |
| --- | --- | --- |
| Delta | Week 1  Baseline  Week 1  Baseline  Week 1 | T_8,_ CP_6_, FC_6_, F_8_, P_8_, C_4_, O_2_, F_4_  F_7_  P_3_  O_2_, P_8_, FC_5_  F_7,_ O_1_, FC_2_, P_z_ |
| Theta | Baseline  Week 1  Baseline  Week 1  Baseline  Week 1  Baseline  Week 1  Baseline  Week 1  Baseline  Week 1 | Fp_2_  FC_2_  P_8_  P_4_  P_4_, O_1_  T_8_  F_7_, F_8_  Fp_2_  O_z_  FC_1_, P_z_, P_7_, C_z_  O_2_  P_8_, T_7_, C_4_, CP_6_ |
| Alpha_1_ | Baseline  Week 1  Baseline | F_7_, F_8_, Fp_1_, F_3_, FC_5_, P_7_, Fp_2_, P_3_  P_z_  O_2_, FC_6,_ F_4_, F_z_, FC_1_ |
| Alpha_2_ | Week 1  Baseline  Week 1  Baseline  Week 1  Baseline  Week 1  Baseline  Week 1  Baseline | O_2_  P_8_  P_4_, O_z_, P_3_  CP_6,_ O_2_, FC_5_, C_4_, F_4_  P_8_  P_4_  O_2_  P_z_, F_3_, C_z_, FC_1_, O_z_,  P_z_,  T_7_ |
| Beta | Week 1  Baseline  Week 1  Baseline  Week 1  Baseline  Week 1  Baseline  Week 1  Baseline  Week 1  Baseline | F_z_  T_7_  FC_2_  P_z_, P_3_  CP_5_  FC_1_  C_3_, FC_6_  CP_6_  P_4_  FC_6_, FC_5_, O_2_  Fp_1_  P_4_, O_z_ |

**Supplementary Figure 3.** Receiver operator curve (ROC) & area under the curve (AUC) scores for each of the scalp-localized EEG bands (EEG power at delta [A], theta [B], alpha_1_ [C], alpha_2_ [D], beta [E]) using Random Forest.


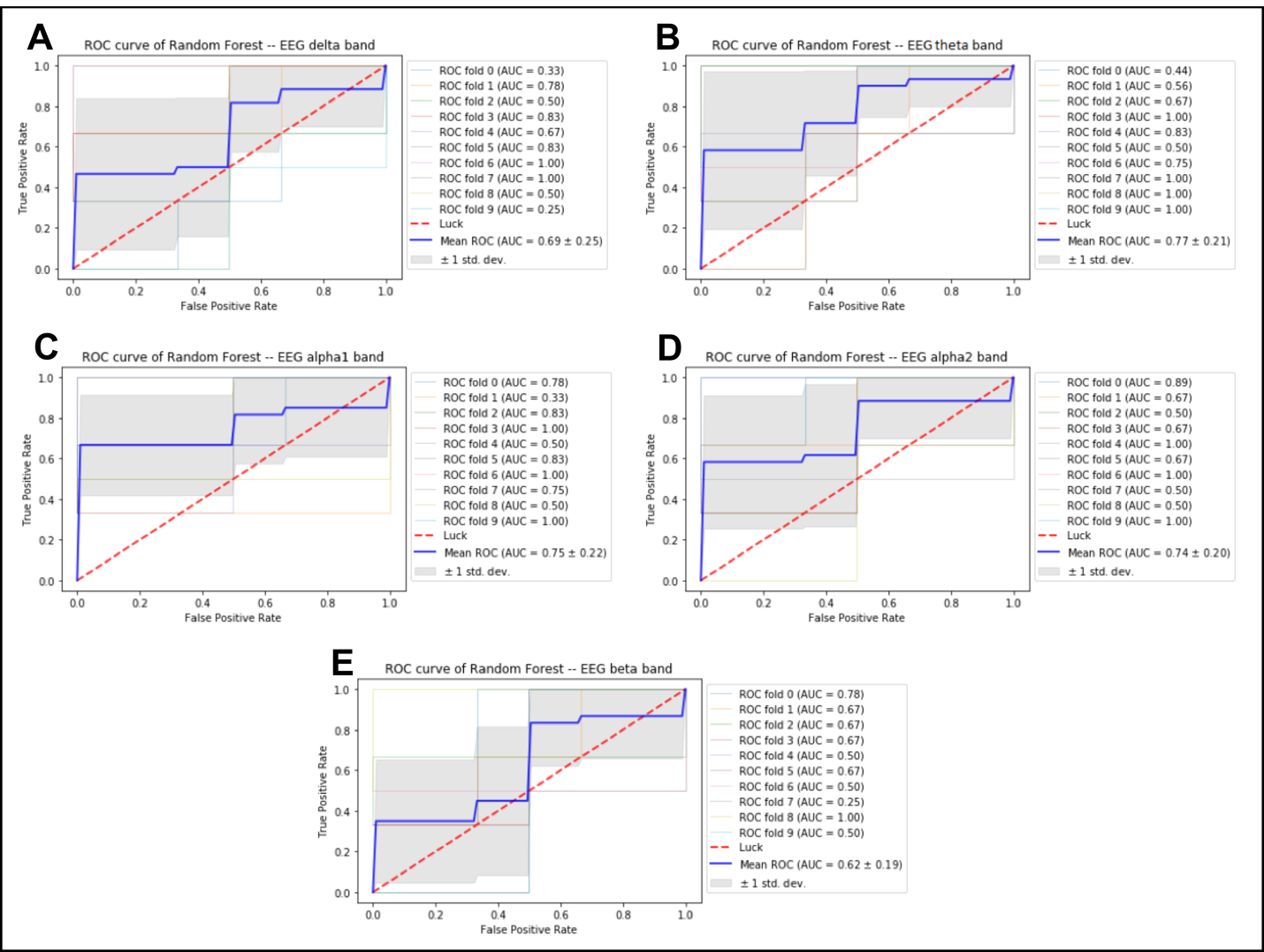


**Supplementary Table 4.** Clinical and demographic features at baseline and week 1 (as well as changes in clinical features) which were predictive of antidepressant response. Features are organized based on importance (the most predictive feature listed first and least predictive listed last).

| **Predicative Features** | **Description** |
| --- | --- |
| MADRS #6 Week 1 | Concentration difficulties at week 1 |
| MADRS #2 Change | Reported sadness change score (baseline to week 1) |
| MADRS Total Baseline | MADRS total at baseline |
| MADRS #6 Change | Concentration difficulties change score (baseline to week 1) |
| MADRS #1 Week 1 | Reported sadness at week 1 |
| MADRS #5 Week 1 | Reduced appetite at week 1 |
| MADRS #5 Baseline | Reduced appetite at baseline |
| MADRS #9 Change | Pessimistic thoughts change score (baseline to week 1) |
| MADRS #5 Change | Reduced appetite change score (baseline to week 1) |
| MADRS Total Change | MADRS total change score (baseline to week 1) |
| MADRS #10 Change | Suicidal thoughts change score (baseline to week 1) |
| MADRS #2 Week 1 | Reported sadness at week 1 |
| MADRS #4 Change | Reduced sleep change score (baseline to week 1) |
| MADRS #3 Baseline | Inner tension at baseline |
| MADRS #6 Baseline | Concentration difficulties at baseline |
| MADRS #10 Week 1 | Suicidal thoughts week 1 |
| MADRS Total Week 1 | MADRS total at week 1 |
| MADRS #8 Week 1 | Inability to feel at week 1 |
| MADRS #3 Change | Inner tension change score (baseline to week 1) |
| MADRS #8 Change | Inability to feel change score (baseline to week 1) |
| Sex | Male/Female |
| MADRS #9 Baseline | Pessimistic thoughts at baseline |
| MADRS #4 Baseline | Reduced sleep at baseline |
| MADRS #9 Week 1 | Pessimistic thoughts at week 1 |
| MADRS #2 Baseline | Reported sadness at baseline |
| Age | Years |
| MADRS #7 Change | Lassitude change score (baseline to week 1) |
| MADRS #8 Baseline | Inability to feel at baseline |
| MADRS #1 Change | Apparent sadness change score (baseline to week 1) |
| MADRS #10 Baseline | Suicidal thoughts at baseline |
| MADRS #7 Week 1 | Lassitude at week 1 |

**Supplementary Figure 4.** Receiver operator curve (ROC) & area under the curve (AUC) scores for clinical/demographic features using Random Forest.


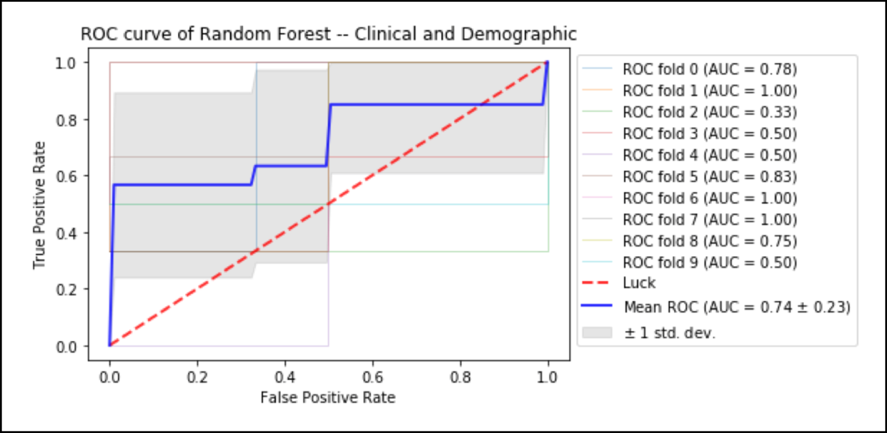


**Supplementary Table 5.** Summary of the best parameter values that were obtained for each ML method derived using Exhaustive Grid Search (implemented as a GridSearchCV function in scikit-learn v0.19.2 library)

|  | **Parameter values** | **Experiment A** | **Experiment B** | **Experiment C** | **Experiment D** | **Experiment E** |
| --- | --- | --- | --- | --- | --- | --- |
| **Random Forest** | No. of estimators | 100 | 101 | 100 | 100 | 105 |
|  | Criterion | Gini impurity | Gini impurity | Gini impurity | Gini impurity | Gini impurity |
|  | Splitter | Best & Radom split | Best split | Best & Radom split | Best & Radom split | Best split |
|  | Max_depth | 2 | 1 | 2 | 2 | 3 |
|  | Min_samples_split | 2 | 2 | 2 | 1 | 3 |
|  | Min_samples_leaf | 1 | 1 | 1 | 1 | 1 |
|  | Max_features | 2 | 2 | 2 | 2 | 3 |
|  | Max_leaf_nodes | None | None | None | None | None |
|  | Min_impurity_decrease | $\frac{N_{t}}{N} \times(Impurity- \frac{N_{r}}{N} \times Right Impurity- \frac{N_{l}}{N} \times left Impurity)$  Where $N$ is the total number of samples, $N_{t}$ is the number of samples at the certain node, $N_{r}$ and $N_{l}$ are the number of samples in the right and left child nodes. | | | | |
|  | Use Bootstrap samples to build trees | True | True | True | True | True |
|  | Use out-of-bag samples to estimate generalization accuracy | True | True | True | True | True |
|  | n_jobs | -1 | -1 | -1 | -1 | -1 |
|  | Verbosity when fitting & predicting | 0 | 0 | 0 | 0 | 0 |
|  | Warm_start to reuse the solution of the previous call to fit & add more estimators to ensemble | False | False | False | False | False |
|  | class_weight | balanced | balanced | balanced | balanced | balanced |
| **Adaboost** | Base_estimator used to build boosted ensemble | None | None | None | None | None |
|  | n_estimators | 53 | 115 | 98 | 100 | 100 |
|  | Learning_rate | 1 | 1.3 | 2 | 1 | 1.6 |
|  | Algorithm | Discrete boosting | | | | |
|  | Random_state | None | None | None | None | None |
| **Support Vector Machine (SVM)** | Penalty parameter C of the error | 1000 | 1000 | 100 | 100 | 1000 |
|  | Kernel | Radial Basis Function | | | | |
|  | Kernel coefficient (parameter gamma) | 0.001 | 0.01 | 0.01 | 0.01 | 0.01 |
|  | Use the shrinking heuristic | True | True | True | True | True |
| **Classification & Regression Tree (CART)** | Criterion | Gini impurity | Gini impurity | Gini impurity | Gini impurity | Gini impurity |
|  | Splitter | Best split | Best split | Best/random split | Best split | Best split |
|  | Max_depth | 2 | 2 | 2 | 2 | 3 |
|  | Min samples split | 2 | 4 | 3 | 2 | 3 |
|  | Min_samples_leaf | 1 | 1 | 1 | 1 | 1 |
|  | Max_features | 2 | 2 | 2 | 2 | 2 |
|  | Random_state | None | None | None | None | None |
|  | Max_leaf_nodes | None | None | None | None | None |
|  | Min_impurity_decrease | $\frac{N_{t}}{N} \times(Impurity- \frac{N_{r}}{N} \times Right Impurity- \frac{N_{l}}{N} \times left Impurity)$  Where $N$ is the total number of samples, $N_{t}$ is the number of samples at the certain node, $N_{r}$ and $N_{l}$ are the numbers of samples in the right and left child nodes. | | | | |
| **Multilayer Perceptron (MLP)** | Hidden_layer_sizes  Activation | 100  Relu/ sigmoid | 67  Relu/ sigmoid | 88  Relu/ sigmoid | 100  Relu/ sigmoid | 74  Relu/ sigmoid |
|  | Solver for weight optimization | A stochastic gradient-based optimizer (‘adam’ algorithm) | | | | |
|  | L2 penalty (regularization term) parameter | 0.0001 | 0.001 | 0.0007 | 0.00033 | 0.00021 |
|  | Learning_rate | 0.001 | 0.006 | 0.001 | 0.001 | 0.009 |
|  | Maximum number of iterations | 200 | 200 | 200 | 200 | 200 |
|  | Shuffle samples in each iteration | True | True | True | True | True |
|  | Random_state | None | None | None | None | None |
|  | Warm_start | False | False | False | False | False |
|  | Value for numerical stability in adam | 1e-7 | 1e-7 | 1e-7 | 1e-7 | 1e-7 |
|  | Validation_fraction of training data | 0.3 | 0.3 | 0.3 | 0.3 | 0.3 |
|  | Exponential decay rate for estimates of first moment vector in adam | 0.004 | 0.008 | 0.007 | 0.001 | 0.0026 |
| **Gaussian Naive Bayes** | Prior probabilities of the classes | None (default) | | | | |
|  | Smoothing variable: represents the portion of the largest variance of all features that is added to variances for calculation stability | 1e-9 (default) | | | | |

**Supplementary Table 6.** Dimensionality of data matrices.

|  | **Analytical base tables (ABT)** | | **After applying the feature importance method** | | **After applying the feature mapping method** | |
| --- | --- | --- | --- | --- | --- | --- |
|  | **# Samples** | **# Features** | **# Samples** | **# Important features** | **# Samples** | **# Mapped features** |
| **Experiment A**  ***sLORETA Delta***  ***sLORETA Theta***  ***sLORETA Alpha_1_***  ***sLORETA Alpha_2_***  ***sLORETA Beta*** | 51 per band | 168 per band (84 ROIs & 2 time points) | 51 per band | Delta: 6  Theta: 10  Alpha_1_: 13  Alpha_2_: 9  Beta: 12 | 51 per band | 2 (responders & non-responders) |
| **Experiment B**  ***Delta EEG***  ***Theta EEG***  ***Alpha_1_ EEG***  ***Alpha_2_ EEG***  ***Beta EEG*** | 51 per band | 56 per band (28 electrodes & 2 time points) | 51 per band | Delta: 17  Theta: 20  Alpha_1_: 14  Alpha_2_: 20  Beta: 17 | 51 per band | 2 (responders & non-responders) |
| **Experiment C**  ***Age***  ***Sex***  ***MADRS Scores*** | 51 | 35  (age, sex, individual/ total MADRS at 2 time points & change scores) | 51 | 31 | 51 | 2 (responders & non-responders) |
| **Experiment D**  **(*All important features + cordance at 2 time points*)** | 51 | 273 | NA | NA | 51 | 2 (responders & non-responders) |
| **Experiment E**  **(*All important features + cordance at 2 time points*)** | 51 | 273 | 51 | 12 | 51 | 2 (responders & non-responders) |
