# Supplementary material for: Leveraging Machine Learning Approaches for Predicting Antidepressant Treatment Response Using Electroencephalography (EEG) and Clinical Data
Source: Front Psychiatry. 2019 Jan 14;9:768. doi: 10.3389/fpsyt.2018.00768 (PMC6339954; doi:10.3389/fpsyt.2018.00768)
Supplement: Supplementary file 2 [file Data_Sheet_2.docx]

**SUPPLEMENTARY INFORMATION**

**Machine Learning Details:**

**1. CART:**

For CART classification splitting, we applied the best random split based on entropy, which is the most commonly used information gain function to measure the quality of the split. With respect to maximum tree depth, the nodes were expanded until all leaves were pure or until all leaves contained less than the minimum number of samples. To avoid overfitting, we applied the following regularization methods to prune the tree: (a) The minimum number of samples was assigned a value = 2; (b) The number of features to consider when looking for the best split was chosen to be equal to sqrt(number of features)

**2. SVM:**

We first considered a set of kernels, namely, the linear, polynomial, radial basis function, sigmoid, and gaussian for SVM. The hyper-parameters of these kernels were tuned using the Exhaustive Grid Search (implemented as a [GridSearchCV](http://scikit-learn.org/stable/modules/generated/sklearn.model_selection.GridSearchCV.html#sklearn.model_selection.GridSearchCV) function in scikit-learn v0.19.2 library). Overall, we obtained the best SVM results when using the radial basis function kernel with the parameters ‘gamma’=0.01 (out of {0.1,0.01,0.001}) and ‘C’=1000 (out of {1,10,100,1000,10000}). These were used in the current manuscript. The behavior of the model is sensitive to the gamma parameter. This means that if gamma is too large, the radius of the area of influence of the support vectors only includes the support vector itself and no amount of regularization with C will be able to prevent overfitting. Conversely, when gamma is very small, the model is too constrained and cannot capture the shape of the data. The region of influence of any selected support vector would include the whole training set. Thus, this intermediate value of the gamma parameter implicitly showed that the SVM could be generalized well by selecting a larger number of support vectors (i.e., larger C values).

**3. Gaussian Naive Bayes (GNB):**

GNB is an extension of naïve Bayes, a parametric classification technique that uses Bayes probability to make predictions. GNB first calculates the Gaussian likelihood probability of a patient to be a responder, given the set of features. It then classifies the patient as responder when the computed likelihood probability is greater than a certain threshold (e.g., 0.5); otherwise it identifies the patient as non-responder.

**4. Adaboost:**

Adaboost is a general ensemble predictive method that builds a strong classifier by using several weak classifiers. That is, it first builds a weak classifier from (training) data, and it boosts it by creating a second classifier that attempts to correct the prediction errors from the first one. It sequentially creates classification models until no further improvement in the prediction behavior is obtained or when a maximum number of classifiers is reached. It then predicts a patient to be a (test data) responder or non-responder by calculating the weighted average of predictions that are obtained from the created weak classifiers.

**5. Multilayer Perceptron (MLP):**

MLP is an architecture of feedforward artificial neural networks. It consists of at least three layers of nodes called input, hidden and output layers. Each node, except for input, is a ‘neuron’ that uses a nonlinear [activation function](https://en.wikipedia.org/wiki/Activation_function). These nodes are also interconnected by weights (or parameters). By utilizing a [supervised learning](https://en.wikipedia.org/wiki/Supervised_learning) technique called [backpropagation](https://en.wikipedia.org/wiki/Backpropagation), we can learn (or estimate) the parameters of MLP using the patients in the training data. Once MLP is learned, it can take any patient (in test data) through the input layer, and classifies it as a responder or non-responder based on a decision function (e.g., logistic function) at the output layer.
